# Molecular identification and functional characterization of two glycosyltransferases genes from *Fallopia multiflora*

**DOI:** 10.3389/fpls.2022.1017122

**Published:** 2022-12-06

**Authors:** Qizhong Cai, Changzheng Liu, Lu Liu, Yuewei Ge, Xuanxuan Cheng, Bi Luo, Liangyun Zhou, Quan Yang

**Affiliations:** ^1^ School of Traditional Chinese Medicine, Guangdong Pharmaceutical University, Guangdong Provincial Research Center on Good AgriculturalPractice and Comprehensive Agricultural Development Engineering Technology of Cantonese Medicinal Materials, Comprehensive Experimental Station of Guangzhou, Chinese Material Medica, China Agriculture Research System (CARS-21-16), Guangzhou, China; ^2^ Key Laboratory of State Administration of Traditional Chinese Medicine for Production & Development of Cantonese Medicinal Materials, Guangzhou, China; ^3^ State Key Laboratory Breeding Base of Dao-di Herbs, National Resource Center for Chinese Materia Medica, China Academy of Chinese Medical Sciences, Beijing, China; ^4^ School of Traditional Chinese Medicine, Guangdong Pharmaceutical University, Guangzhou, China

**Keywords:** *Fallopia multiflora* (Thunb.) Harald., glycosylation, uridine-diphosphate glycosyltransferase, metabolic engineering, transgenic hairy roots

## Abstract

The traditional Chinese medicine plant *Fallopia multiflora* (Thunb.) Harald. contains various pharmacodynamically active glycosides, such as stilbene glycosides, anthraquinone (AQ) glycosides, and flavonoid glycosides. Glycosylation is an important reaction in plant metabolism that is generally completed by glycosyltransferase in the last step of the secondary metabolite biosynthesis pathway, and it can improve the beneficial properties of many natural products. In this study, based on the transcriptome data of *F. multiflora*, we cloned two Uridine-diphosphate-dependent glycosyltransferases (UGTs) from the cDNA of *F. multiflora* (*FmUGT1* and *FmUGT2*). Their full-length sequences were 1602 and 1449 bp, encoding 533 and 482 amino acids, respectively. *In vitro* enzymatic reaction results showed that *FmUGT1* and *FmUGT2* were promiscuous and could catalyze the glycosylation of 12 compounds, including stilbenes, anthraquinones, flavonoids, phloretin, and curcumin, and we also obtained and structurally identified 13 glycosylated products from both of them. Further experiments on the *in vivo* function of *FmUGT1* and *FmUGT2* showed that 2, 3, 5, 4’- tetrahydroxy stilbene-2-*O-β-d*-glucoside (THSG) content in hairy roots was elevated significantly when *FmUGT1* and *FmUGT2* were overexpressed and decreased accordingly in the RNA interference (RNAi) groups. These results indicate that *FmUGT1* and *FmUGT2* were able to glycosylate a total of 12 structurally diverse types of acceptors and to generate *O*-glycosides. In addition, *FmUGT1* and *FmUGT2* efficiently catalyzed the biosynthesis of THSG, and promoted the production of AQs in transgenic hairy roots.

## Introduction

As a perennial herb in the *Polygonaceae* family, *Fallopia multiflora* is generally used in traditional Chinese medicine and contains various major active components including stilbenes, anthraquinones (AQs), flavonoids, and polysaccharides (Bi et al., 2020; Wang et al., 2021). The herb has a wide range of effects in clinical and therapeutic care, and previous research on its active components has focused on 2, 3, 5, 4’- tetrahydroxy stilbene-2-*O-β-d*-glucoside (THSG) and AQ glucosides. Researches suggested that THSG may be the attractive candidate for Parkinson’s disease (Zhang et al., 2017) and could enhance the learning and memory (Wang et al., 2011), it also has the anti-atherosclerotic properties (Yao et al., 2015) and neuroprotective effects (He et al., 2015). Furthermore, the AQ glucosides may have potential anti-cancer properties (Kwon et al., 2009; Sun et al., 2015) and anti-aging effect (Lo et al., 2015). Given that there are various glycosides in *F. multiflora*, there must be some corresponding glycosyltransferases related to the synthesis pathway of THSG, AQ glucosides, and even other glycosides in *F. multiflora* (Xia et al., 2018), and in this paper we set out to identify them and characterize their catalytic properties. Our previous study has revealed 44 candidate GT genes in the transcriptome of *F. multiflora* and we initially found seven of them have the glycosylation activity. In this study, we selected two *FmUGTs* with high catalytic activity and promiscuity to further explore their roles *in planta* and in the biosynthesis pathway of THSG and AQs, and named them *FmUGT1* and *FmUGT2*, respectively.

Plant secondary metabolites have been shown to exhibit a variety of biological activities (Fang et al., 2022; Lin et al., 2022). However, some of this effectiveness is always limited by low water solubility and stability due to specific chemical structure, but glycosylation could eliminate these problems (Plaza et al., 2014). Glycosyltransferases (GTs; EC 2.4.x.y) have a large supergene family and are found in many types of organisms (De Bruyn et al., 2015). These enzymes can catalyze the formation of glycosyl bonds between glycosyl donors and specific acceptors with high efficiency and stereoselectivity, and could always improve the stability, solubility, and bioavailability of some resultant compounds (Dan et al., 2004; Jiang et al., 2008; Li et al., 2020). Nucleotide-activated sugar or phosphate glycoside can be used as the glycosyl donor, and acceptors include macromolecules such as proteins, lipids, cell wall polysaccharides, and even small molecules such as secondary metabolites in plants or other microorganisms (Lim, 2005; De Bruyn et al., 2015).

The overexpression (OE) of *GTs* gene in plants can also increase the content of plant secondary metabolites. Ma et al. (2007) found that OE of the *UGT73B6* in *Rhodiola sachalinensis* increased the conversion rate of tyrosol aglycon to salidroside, showing that UGT73B6 might be the key enzyme involved in salidroside biosynthesis. Tan et al. (2022) also found that OE of IiUGTs could increase lignan glycosides contents in hairy roots, which indicated that *IiUGTs* play an important role in the biosynthetic route of lignan glycosides in *I. indigotica*. Moreover, the OE of *ZmCGT1* could induce accumulation of isoorientin in maize silk (Sun et al., 2022). These results not only provide a theoretical basis for the study of biosynthesis pathways but also provide technical guidance for the use of OE to enhance the content of secondary metabolites in plants.

Xia et al. (2018) have studied the THSG biosynthetic pathway and validated the enzymatic activities responsible for the resveratrol synthesis, hydroxylation, and glycosylation reactions involved in THSG biosynthesis *in vitro*, and found that GTs in *F. multiflora* could catalyze the glycosylation of hydroxylated resveratrol to form THSG. In this study, we performed prokaryotic expression, protein purification, and enzymatic product identification studies on *FmUGT1* and *FmUGT2* and not only discovered novel tool enzymes that promoted the development of different compounds’ glycosylation but also provided important results in the study of THSG the biosynthesis pathway. Additionally, we also constructed a glycosides-rich transgenic system by regulating the transcription levels of *FmUGT1* and *FmUGT2* in the hairy roots of *F. multiflora*, which provided a production platform for the biosynthesis of THSG and AQs.

## Methods and materials

### Plant materials

Stems, leaves, and roots of *F. multiflora* were collected from Deqing County, Zhaoqing City, Guangdong Province, China (23° 20′ 2′′ N, 112° 14′ 16′′ E, altitude 60 m, autumn, 2018). These materials were well cleaned and dried then flash-frozen with liquid nitrogen and stored at -80°C. The seeds of *F. multiflora* were obtained from the same location and were sterilized using 0.1% mercuric chloride for 18 min, after which the seeds were flushed thoroughly with sterile distilled water and germinated in MS solid medium [pH=5.8; (Murashige and Skoog, 1962)] at 25°C in the dark until germination. The germinated seeds then grew at 25°C with a light intensity of 1500 LX provided by cool white fluorescent lamps during the light period (16 h light/8 h dark) to produce *F. multiflora* seedlings. Transgenic hairy roots were cultured in solid MS medium at 25°C in the dark, and the identified transgenic hairy root lines that grew stably were transferred into liquid MS medium and cultured at 130 rpm using a light avoiding shaker.

### Cloning of *FmUGT1* and *FmUGT2* from *F. Multiflora*

In this study, we selected two *GT* genes based on the *F. multiflora* transcriptome data and named them *FmUGT1* and *FmUGT2* (GenBank accession numbers: ON262204 and ON262205, respectively). Their specific amplification primers are shown in Table S1. Total RNA of *F. multiflora* stems, leaves, and roots were extracted with RNAprep Pure Plant Plus Kit (Polysaccharides & Polyphenolics-rich; Tiangen, Beijing, China). The total RNA reverse transcription was completed using PrimeScript™ II 1^st^ Strand cDNA Synthesis Kit (TaKaRa, Dalian, China), and KOD-Plus-Neo (TOYOBO, Osaka, Japan) was used for full-length cDNA cloning of the *FmUGT1* and *FmUGT2*. We used *pEASY* ^®^-Uni Seamless Cloning and Assembly Kit (Transgene, Beijing, China) to subclone the target sequences into pET-32a at *BamH* I site to construct the pET-32a-*FmUGT1* and pET-32a-*FmUGT2* recombinant expression vectors.

### Bioinformatics analysis of *FmUGT1* and *FmUGT2*

ExPASY Proteomics Server program ProtParam (Gasteiger et al., 2005) was used to analyze the physicochemical properties of the proteins encoded by *FmUGT1* and *FmUGT2*; Cell-PLOC 2.0 [http://www.csbio.sjtu.edu.cn/bioinf/Cell-PLoc-2/; (Chou and Shen, 2010)] was used to predict the subcellular localization of *FmUGT1* and *FmUGT2*; Transmembrane domains were predicted using SignalP 5.0 [https://services.healthtech.dtu.dk/service.php/SignalP-5.0; (Armenteros et al., 2019)];The secondary structure of *FmUGT1* and *FmUGT2* were predicted by EXPASY-SOPMA [https://web.expasy.org/protparam/; (Gasteiger et al., 2005)]; Three-dimensional structure models of *FmUGT1* and *FmUGT2* were constructed *via* SWISS-MODEL [https://swissmodel.expasy.org/; (Kelley and Sternberg, 2009)]; We used DNAMAN software [version 8.0; (Naresh et al. (2017)] for multiple sequence alignment and homology comparison with corresponding amino acid sequences of other species, and MEGA software [version 6.0; (Tamura et al., 2013)] for neighbor-joining phylogenetic tree construction. Finally, we calculated evolutionary distances by Poisson correction with 1000 bootstrap replications.

### Induction and purification of *FmUGT1* and *FmUGT2* recombinant protein

*Trans1*-T1 Phage Resistant Chemically Competent Cells (Transgene, Beijing, China) were used as cloning hosts and *Transetta* (DE3) Chemically Competent Cells (Transgene, Beijing, China) were used to express pET-32a-*FmUGT1* and pET-32a-*FmUGT2*. The transformed DE3 cells were cultured with final concentration of isopropyl *β-d*-1-thiogalactopyranoside (0.4 mM; IPTG) at 16°C for 20 h after OD_600_ reached 0.6- 0.8 to induce pET-32a-*FmUGT1* and pET-32a-*FmUGT2* recombinant protein. The cell lysate was obtained using an ultrasonic crusher (Sonic & Materials, Inc., Newtown, CT, USA) at 30 kHz for 5 min in a water-ice bath, then centrifuged at 12,000 rpm for 15 min at 4°C. The supernatant was collected as crude enzyme and mixed well with 6 × protein loading buffer (Transgene, Beijing, China) and put into a boiling water bath for 10 min. The protein itself was subjected to 10% sodium dodecyl sulphate–polyacrylamide gel electrophoresis (SDS-PAGE) for detection.

After this, the recombinant protein was eluted with 0.05 M Tris-HCl buffer (pH = 7.4) containing 50-500 mM imidazole (the concentration gradient was set to every 50 mM) sequentially from low to high concentrations in a Ni-NTA column, and the eluate was collected. The eluate was proportionally added to 6 × protein loading buffer (transgene, Beijing, China) and put into a boiling water bath for 5 min for 10% SDS-PAGE detection. Next, the purified proteins were concentrated and desalinized with the Amicon ultra-15 (ultracel-50 regenerated cellulose membrane, 15 ml sample volume; Merck, Germany) centrifugal filter column, and we measured the concentration using by the *Easy* protein quantitative Kit (Bradford; transgene, Beijing, China).

### Catalytic parameters analysis and identification of the structure of the glycosylated product

For the catalytic parameters analysis, using THSG and emodin as the substrate for FmUGT1 and FmUGT2, respectively. Each reaction containing final concentration of 200 μM substrate and 400 μM uridine-diphosphate glucose (UDPG), and 50 μg purified FmUGTs protein, then was filled up with 0.05 M Tris-HCl buffer. The optimum reaction temperature was between 4 to 70°C, and the optimum pH value was between 4.0 and 10.8. Citric acid-sodium citrate buffer, Tris-HCl buffer, and Na_2_CO_3_-NaHCO_3_ buffer were used for pH ranges of 4.0- 7.0, 7.0- 9.0, and 9.0- 11, respectively. Final concentration of 5 mM different metal cations (BaCl_2_, CaCl_2_, CoCl_2_, CuCl_2_, FeCl_2_, MgCl_2_, MnCl_2_, ZnCl_2_) were added into the reaction to measure the effect of metal cations on the enzyme activity. For the kinetic studies, 60 μg purified FmUGTs were incubated in the volume of 300 μL with 0.8 mM UDP-glucose, and at least 7 substrate concentrations between 5 to 480 μM were set. The kinetics parameters were analyzed using Lineweaver-Burk plots.

**Figure 1:**
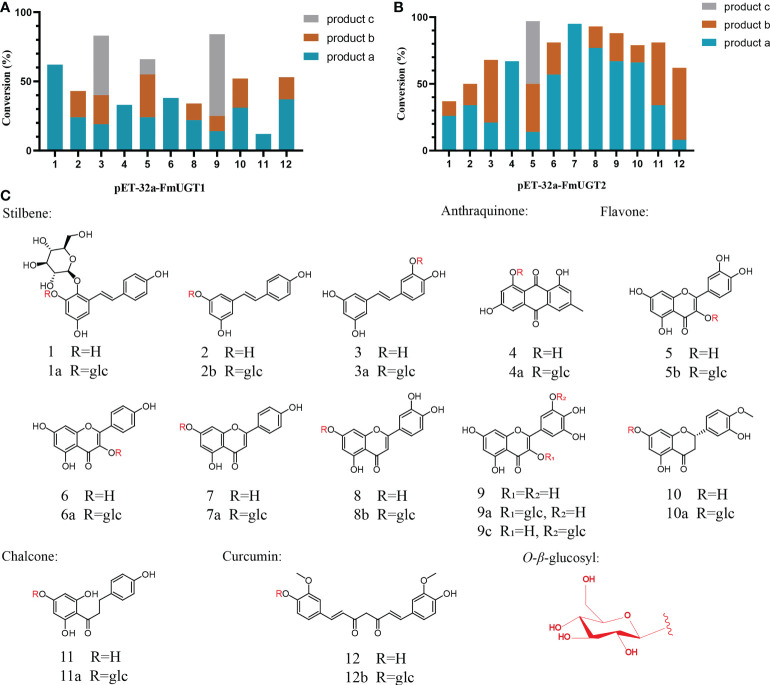
Substrate promiscuity of *FmUGT1* and *FmUGT2*. **(A)** Conversion rates (%) of *FmUGT1*. **(B)** Conversion rates (%) of *FmUGT2*. **(C)** Structures of substrates 1-12, (1) 2, 3, 5, 4’ -Tetrahydroxy stilbene-2-*O-β-d*-glucoside (THSG); (2) Resveratrol; (3) Piceatannol; (4) Emodin; (5) Quercetin; (6) Kaempferol; (7) Apigenin; (8) Luteolin; (9) Myricetin; (10) Hesperetin; (11) Phloretin; (12) Curcumin. (1a) 2, 3, 5, 4’- tetrahydroxy-stilbene-2, 3-di*-O-β-d*-glucoside (polygonimitin C); (2a) Resveratrol 3-*O-β-d*-glucoside; (3a) Piceatannol 3’-*O-β-d*-glucoside; (4a) Emodin 8-*O-β-d*-glucoside; (5b) Quercetin 3-*O-β-d*-glucoside; (6a) Kaempferol 3-*O-β-d*-glucoside. (7a) Apigenin 7-*O-β-d*-glucoside; (8a) Luteolin 7-*O-β-d*-glucoside; (9a) Myricetin 3-*O-β-d*-glucoside; (9c) Myricetin 5’-*O-β-d*-glucoside; (10a)Hesperetin 7-*O-β-d*-glucoside; (11a) Phloretin 4’-*O-β-d*-glucoside; (12b) Curcumin 4’-*O-β-d*-glucoside.

In total 12 compounds including stilbenes, flavonoids, AQs, phloretin, and curcumin (obtained from Macklin Biochemical Co., Ltd, China; **Figure 1**) were used as sugar acceptors for *FmUGT1* and *FmUGT2*. Enzyme assay was performed in 200 μL reactions containing 50 μg recombinant protein, final concentration of UDPG (400 μM) and substrate (200 μM), then was filled up with 0.05 M Tris-HCl buffer (pH=7.4). The reaction was placed at 30°C for 12 h and then terminated by mixing with 400 μL of cold methanol. The reaction was separated by a 15 min centrifuge at 13,000 rpm, and the supernatant was collected and filtered through a 0.45 μm nylon filter (Jinlong, China). We carried out our analysis of reaction products on a Waters 1525 HPLC system with a Waters Symmetry C_18_ Column at 30°C, and the mobile phase condition and gradient program are shown in Table S2.

Our LC-MS/MS analysis was performed by AB Sciex 5600 Q-TOF-MS with the ACQUITY UPLC BEH C_18_ column (100 mm × 2.1 mm I.D., 1.7 μm, Waters Co., Ltd., USA); and the mobile phase condition and gradient program are shown in Table S3. The optimized ESI source parameters were set as follows: spray voltage, 4.5 kV (negative ion mode); sheath gas flow rate, 20 (arbitrary units); auxiliary gas flow rate, 5 (arbitrary units); capillary temperature, 500°C; and Collision-induced dissociation voltage, 25 V.

Next the reaction volume was enlarged to 30 mL containing 2 mM of UDPG, and 1 mM of sugar acceptors and filled up with crude FmUGT1 and FmUGT2 enzyme to 30 mL for the accumulation of glycosylation products. After incubation at 30°C for 12h, the reaction solution was extracted five times with twice its volumes of ethyl acetate. The ethyl acetate layers were combined and dried using a rotary evaporator, then redissolved with 2 mL methanol and filtered using a 0.45 μm nylon filter (Jinlong, China). The filtrate was then separated on a CXTH LC3000 semi-HPLC system with a cosmosil 5C_18_-MS-II (250 mm × 10 mm I.D., 5 μm, Nacalai Tesque, Japan), and the gradient program is shown in Table S4. ^1^H- nuclear magnetic resonance (NMR) spectroscopy and ^13^C-NMR spectroscopy were used to identify the separated glycosylation products.

### Structural characterization of the glycosylated product

(1) compound 1a (6.7 mg, brown amorphous solid): Polygonimitin C:
ESI-MS *m/z* 567.1730 [M-H]^-^;^1^H-NMR (500 MHz, DMSO-*d_6_*) δ 7.50 (1H, d, *J* = 16.5 Hz, H-α), 6.88 (1H, d, *J* = 16.5 Hz, H-β), 6.70 (1H, d, *J* = 2.4 Hz, H-6), 6.60 (1H, d, *J* = 2.6 Hz, H-6), 6.75 (2H, dd, *J* = 1.8, 8.6 Hz, H-3, 5), 7.39 (2H, dd, *J* = 2, 8.4 Hz, H-2, 6), 4.63 (1H, d, *J* = 7.7 Hz, H-1’’). ^13^C-NMR (126 MHz, DMSO-*d_6_*) δ 157.1 (C-4’), 154.0 (C-5), 151.1 (C-11b), 136.6 (C-1’’), 132.4 (C-3), 128.6 (C-1), 128.5 (C-β), 128.0 (C-2’, 6’), 121.0 (C-α), 115.5 (C-3’, 5’), 104.7 (C-1’’), 104.0 (C-6), 103.6 (C-1’’’), 102.1 (C-4), 77.1 (C-5’’), 77.0 (C-5’’’), 76.2 (C-3’’), 75.7 (C-3’’’), 74.2 (C-2’’), 73.4 (2’’’), 70.0 (C-4’’), 69.6 (C-4’’’), 61.00 (C-6’’), 60.6 (C-6’’’) (Zhou et al., 1994).
(2) compound 3a (3.7 mg, brown amorphous solid): Piceatannol 3’-*O-β-d*-glucoside
ESI-MS *m/z* 405.1198 [M-H]^-^;^1^H-NMR (500 MHz, DMSO-*d_6_*) δ 7.44 (d, *J* = 16.4 Hz, 1H, H-2’), 7.06 (dd, 1H, H-6’), 6.89 (d, *J* = 11.6 Hz, 1H, H-*α*), 6.85 (d, *J* = 10.8 Hz, 1H, H-β), 6.79 (d, *J* = 9.2 Hz, 1H, H-5’), 6.39 (d, 2H, H-2, 6), 6.12 (t, 1H, H-4), 4.76 (d, 1H, H-1’’), 3.77 - 3.11 (m, 6H, Glc). ^13^C-NMR (126 MHz, DMSO-*d_6_*) δ 158.50 (C-5, 3), 146.70 (C-3’), 145.66 (C-4’), 139.22 (C-1), 128.91 (C-1’), 127.84 (C-β), 126.42 (C-α), 122.13 (C-6’), 115.94 (C-2’), 114.30 (C-5’), 104.45 (C-6), 102.50 (C-4), 101.90 (C-1’’), 77.41 (C-3’’), 76.02 (C-5’’), 73.44 (C-2’’), 70.18 (C-4’’), 60.98 (C-6’’) (Toshiki et al., 1984).
(3) compound 4a (2.1 mg, yellow powder): Emodin 8-*O-β-d*-glucoside:
ESI-MS *m/z* 431.1000 [M-H]^-^; ^1^H-NMR (700 MHz, DMSO-*d_6_*) δ 7.55 (s, 1H, H-4), 7.27 (d, *J* = 2.5 Hz, 1H, H-5), 7.22 (s, 1H, H-2), 6.95 (d, *J* = 2.5 Hz, 1H, H-7), 5.13 (d, *J* = 7.4 Hz, 1H, H-1’’), 3.70 - 3.18 (m, 6H, Glc), 2.44 (s, 3H, CH_3_-3). ^13^C-NMR (176 MHz, DMSO-*d_6_*) δ 190.06 (C-9), 181.25 (C-10), 163.86 (C-6, 8), 161.62 (C-1), 148.60 (C-3), 134.95 (C-11), 132.97 (C-14), 124.31 (C-2), 120.60 (C-4), 113.64 (C-12), 110.89 (C-13), 109.08 (C-5), 108.87 (C-7), 99.94 (C-1’’), 77.28 (C-5’’), 76.23 (C-3’’), 73.11 (C-2’’), 69.50 (C-4’’), 60.55 (C-6’’), 21.60 (CH_3_-3) (He et al., 2019).
(4) compound 6a (3.4 mg, yellow powder): Kaempferol 3-*O-β-d*-glucoside:
ESI-MS *m/z* 447.0950 [M-H]^-^; ^1^H-NMR (500 MHz, Methanol-*d_4_*) δ 8.05 (d, *J* = 8.6 Hz, 2H, H-2’, 6’), 6.88 (d, *J* = 8.7 Hz, 2H, H-3’, 5’), 6.38 (d, *J* = 2.0 Hz, 1H, H-8), 6.19 (d, *J* = 1.9 Hz, 1H, H-6), 5.23 (d, *J* = 7.1 Hz, 1H, H-1’’), 3.70 (dd, *J* = 11.4, 3.9 Hz, 1H, H-6a’’), 3.54 (dd, *J* = 3.3, 2.8 Hz, 1H, H-6b’’), 3.44 (dd, *J* = 9.0 Hz, 1H, H-2’’), 3.39 (dd, *J* = 9.2 Hz, 1H, H-3’’), 3.21 – 3.17 (m, 1H, H-5’’). ^13^C-NMR (126 MHz, Methanol-*d_4_*) δ 179.46 (C-4), 166.58 (C-7), 163.01 (C-5), 161.58 (C-4’), 159.03 (C-8a), 158.55 (C-2), 135.45 (C-3), 132.27 (C-2’, 6’), 122.81 (C-1’), 116.09 (C-3’, 5’), 105.57 (C-4a), 104.17 (C-1’’), 100.11 (C-6), 94.90 (C-8), 78.40 (C-5’’), 75.73 (C-2’’), 71.36 (C-4’’), 62.63 (C-6’’) (Duong et al., 2017).
(5) compound 7a (5.7 mg, pale yellow powder): Apigenin 7-*O-β-d*-glucoside:
ESI-MS *m/z* 431.1017 [M-H]^-^; ^1^H-NMR (500 MHz, DMSO-*d_6_*) δ 12.97 (s, 1H, OH-5), 10.42 (s, 1H, OH-4’), 7.97 (d, *J* = 8.8 Hz, 2H, H-2’, 6’), 6.95 (d, *J* = 8.9 Hz, 2H, H-3’, 5’), 6.87 (d, *J* = 2.1 Hz, 1H, H-3), 6.84 (d, *J* = 2.2 Hz, 1H, H-8), 6.45 (d, *J* = 2.1 Hz, 1H, H-6), 5.41 (d, *J* = 4.9 Hz, 1H, H-1’’), 5.14 (d, *J* = 4.7 Hz, 1H, OH-2’’), 5.07 (d, *J* = 7.3 Hz, 1H, H-3’’), 4.62 (t, *J* = 5.6 Hz, 1H, H-6’’), 3.72 (s, 1H, H-2’’), 3.52 – 3.18 (m, 6H, Glc). ^13^C-NMR (126 MHz, DMSO-*d_6_*) δ 181.99 (C-4), 164.69 (C-7), 162.96 (C-2), 161.35 (C-9), 161.11 (C-4’), 156.94 (C-5), 128.60 (C-2’), 121.03 (C-1’), 116.62 (C-3’), 115.99 (C-5’), 105.33 (C-10), 103.11 (C-3), 99.90 (C-6), 99.52 (C-1’’), 95.26 (C-8), 77.15 (C-3’’), 76.38 (C-5’’), 73.09 (C-2’’), 69.55 (C-4’’), 63.08 (C-6’’) (Ewa, 2020).
(6) compound 8b (4.1 mg, brown amorphous solid): Luteolin 7-*O-β-d*-glucoside:
ESI-MS *m/z* 447.0977 [M-H]^-^; ^1^H NMR (500 MHz, DMSO-*d_6_*) δ 12.99 (s, 1H), 7.45 (d, *J* = 2.2 Hz, 1H, H-2’), 7.43 (s, 1H, H-6’), 6.91 (d, *J* = 8.4 Hz, 1H, H-3’), 6.79 (d, *J* = 2.1 Hz, 1H, H-3), 6.76 (s, 1H, H-3), 6.45 (d, *J* = 2.2 Hz, 1H, H-6), 5.09 (d, *J* = 7.4 Hz, 1H, H-1’’), 3.71 (d, *J* = 10.8 Hz, 1H, H-6β), 3.50 (d, 1H, H-6α), 3.51 – 3.23 (m, 6H, Glc). ^13^C-NMR (126 MHz, DMSO-*d_6_*) δ 181.87 (C-4), 164.43 (C-2), 162.92 (C-7), 161.12 (C-5), 156.94 (C-9), 149.82 (C-4’), 145.69 (C-5’), 121.40 (C-1’), 119.16 (C-2’), 115.95 (C-3’), 113.54 (C-6’), 105.33 (C-10), 99.89 (C-1’’), 99.52 (C-6), 94.72 (C-8), 77.14 (C-3’’), 76.32 (C-5’’), 73.06 (C-2’’), 69.50 (C-4’’), 60.58 (C-6) (Chahra et al., 2018).
(7) compound 9a (3.3 mg, yellow amorphous solid): Myricetin 3-*O-β-d*-glucoside:
ESI-MS *m/z* 479.0887 [M-H]^-^; ^1^H-NMR (500 MHz, DMSO-*d_6_*) δ 12.63 (s, 1H, H-5), 10.91 (s, 1H, H-7), 9.18 (s, 2H, H-3’, 5’), 8.95 (s, 1H, H-4’), 7.20 (s, 2H, H-2’, 6’), 6.38 (d, *J* = 2.1 Hz, 1H, H-8), 6.20 (s, 1H, H-6), 5.48 (s, 1H, H-1’’), 3.60 - 3.11 (m, 6H, Glc). ^13^C-NMR (126 MHz, DMSO-*d_6_*) δ 177.41 (C-4), 164.02 (C-7), 161.25 (C-5), 156.25 (C-2, 9), 145.36 (C-3’, 5’), 136.56 (C-4’), 133.51 (C-3), 120.06 (C-1’), 108.54 (C-2’, 6’), 103.97 (C-10), 100.90 (C-1’’), 98.58 (C-6), 93.31 (C-8), 77.60 (C-3’’), 76.56 (C-5’’), 73.92 (C-2’’), 69.93 (C-4’’), 61.09 (C-6’’) (Khaled et al., 2014).
(8) compound 9c (3.6 mg, yellow amorphous solid): Myricetin 5’-*O-β-d*-glucoside:
ESI-MS *m/z* 431.0891 [M-H]^-^; ^1^H-NMR (500 MHz, DMSO-*d_6_*) δ 12.45 (s, 1H, H-5), 10.79 (s, 1H, H-7), 9.40 (s, 1H, H-4’), 9.31 (s, 1H, H-3’), 8.84 (s, 1H, H-3), 7.54 (d, *J* = 2.2 Hz, 1H, H-2’), 7.53 (d, *J* = 2.2 Hz, 1H, H-6’), 6.47 (d, *J* = 2.0 Hz, 1H, H-8), 6.19 (d, *J* = 2.1 Hz, 1H, H-6), 4.72 (d, *J* = 7.3 Hz, 1H, H-1’’), 3.77 – 3.19 (m, 6H, Glc). ^13^C-NMR (126 MHz, DMSO-*d_6_*) δ 175.82 (C-4), 163.89 (C-7), 160.62 (C-5), 155.60 (C-9), 146.30 (C-2), 145.94 (C-3’), 145.50 (C-4’), 137.31 (C-3), 136.03 (C-1’), 121.01 (C-5’), 110.74 (C-2’), 107.55 (C-10), 102.99 (C-6’), 102.85 (C-1’’), 98.14 (C-6), 93.55 (C-8), 77.21 (C-3’’), 75.85 (C-5’’), 73.28 (C-2’’), 69.58 (C-4’’), 60.59 (C-6’’) (Gu et al., 1997).
(9) compound 10a (2.7 mg, white powder): Hesperetin 7*-O-β-d*-glucoside:
ESI-MS *m/z* 463.1314 [M-H]^-^; ^1^H-NMR (500 MHz, DMSO-*d_6_*) δ 12.04 (s, 1H, OH-5), 9.13 (s, 1H, OH-3’), 6.97 – 6.85 (m, 3H, H-2’, 5’, 6’), 6.16 (t, *J* = 2.0 Hz, 1H, H-6), 6.13 (dd, *J* = 3.9, 2.2 Hz, 1H, H-8), 5.49 (dt, *J* = 12.5, 4.4 Hz, 1H, H-2), 4.97 (dd, *J* = 11.0, 7.6 Hz, 1H, H-1’’), 3.77 (s, 3H, OCH_3_-4’), 3.66 – 3.16 (m, 6H, Glc), 3.16 – 3.09 (m, 1H, H-3α, overlap with the sugar-ring proton signal), 2.76 (ddd, *J* = 17.0, 6.3, 3.1 Hz, 1H, H-3β). ^13^C-NMR (126 MHz, DMSO-*d_6_*) δ 197.09 (C-4), 165.31 (C-7), 162.93 (C-5), 162.63 (C-9), 147.99 (C-4’), 146.48 (C-3’), 130.91 (C-1’), 117.85 (C-6’), 114.17 (C-2’), 111.98 (C-5’), 103.29 (C-10), 99.57 (C-1’’), 96.48 (C-6), 95.49 (C-8), 78.50 (C-2), 77.08 (C-3’’), 76.32 (C-5’’), 73.03 (C-2’’), 69.48 (C-4’’), 60.56 (C-6’’), 55.69 (OCH_3_-4’), 42.19 (C-3) (Shimoda et al., 2008).
(10) compound 11a (3.9 mg, brown amorphous solid): Phloretin 4’-*O-β-d*-glucoside:
ESI-MS *m/z* 435.1352 [M-H]^-^; ^1^H-NMR (500 MHz, DMSO-*d_6_*) δ 7.02 (d, *J* = 8.0 Hz, 2H, H-2, 6), 6.66 (d, *J* = 8.1 Hz, 2H, H-3, 5), 6.04 (s, 2H, H-3’, 5’), 4.87 (d, *J* = 6.8 Hz, 1H, H-1’’), 3.67 – 3.28 (m, 6H, Glc), 3.21 – 3.16 (m, 2H, H-β), 2.77 (d, *J* = 7.9 Hz, 2H, H-α). ^13^C-NMR (126 MHz, DMSO-*d_6_*) δ 205.07 (C=O), 163.80 (C-4’), 163.40 (C-2’), 155.43 (C-4), 131.52 (C-1’), 129.18 (C-3), 115.11 (C-2), 105.30 (C-1), 99.55 (C-1’’), 95.08 (C-5’, 3’), 77.13 (C5’’), 76.44 (C-3’’), 73.06 (C-2’’), 69.45 (C-4’’), 60.51 (C-6’’), 46.30 (C-α), 29.33 (C-β) (Xu et al., 2015).
(11) compound 12b (6.2 mg, brown powder): Curcumin 4’-*O-β-d*-glucoside:
ESI-MS *m/z* 529.1787 [M-H]^-^;^1^H-NMR (500 MHz, DMSO-*d_6_*) δ 7.58 (d, 1H, H-1), 7.56 (d, 1H, H-7), 7.47 – 6.94 (m, 6H, Ph), 6.84 (d, *J* = 7.4 Hz, 1H, H-2), 6.77 (d, 1H, H-6), 6.09 (s, 1H, H-4), 5.00 (d, *J* = 6.6 Hz, 1H, H-1’’), 3.84 (s, 6H, OCH_3_-3’, 3’’), 3.68 - 3.14 (m, 6H, Glc). ^13^C-NMR (126 MHz, DMSO-*d_6_*) δ 185.71 (C-3), 183.77 (C-5), 151.06 (C-4’), 150.78 (C-4’’), 150.10 (C-3’), 149.61 (C-3’’), 142.62 (C-1), 141.52 (C-7), 130.31 (C-1’), 127.89 (C-1’’), 124.84 (C-2), 124.14 (C-6), 124.03 (C-6’), 122.74 (C-6’’) 117.30 (C-5’), 116.67 (C-5’’), 113.00 (C-2’), 112.93 (C-2’’), 102.65 (C-4), 101.23 (C-1’’’), 78.69 (C-2’’’), 78.39 (C-3’’’), 74.71 (C-4’’’), 71.18 (C-5’’’), 62.20 (C-6’’’), 57.37 (OCH_3_-3’), 57.32 (OCH_3_-3’’) (Zhang et al., 2010).

### Construction of plant expression vector

OE primers corresponding to the open reading frame (ORF) of the *FmUGT1* and *FmUGT2* and *att* B adapter from pDONR-221, and the RNAi fragments contained specific 244 and 247 bp fragments of *FmUGT1* and *FmUGT2*, respectively, with *att* B adapter. The primers are shown in **Table S5**. Phusion High-Fidelity DNA Polymerase (Thermo, USA) was used for cloning of the OE fragments and RNAi fragments. Subsequently, we ligated the target fragments into the pDONR-221 entry vector using Gateway BP Clonase™ II Enzyme Mix (Thermo Fisher, USA), and the entry vectors pDONR-*FmUGT1*, pDONR-*FmUGT2*, pDONR-*FmUGT1i*, and pDONR-*FmUGT2i* were validated by sequencing.

We constructed the recombinant OE/RNAi expression vectors with pK7WG2D and pK7GWIWG2D (II) using Gateway LR Clonase™ II Enzyme Mix (Thermo Fisher, USA) and validated the recombinant expression vectors pK7WG2D-*FmUGT1*, pK7WG2D-*FmUGT2*, pK7GWIWG2D-*FmUGT1i* and pK7GWIWG2D-*FmUGT2i* by PCR analysis and sequencing. These recombinant expression vectors were then transformed into the *Agrobacterium rhizogenes* (C58C1) by liquid nitrogen freeze-thawing to induce the production of *F. multiflora* transgenic hairy roots.

### Genetic transformation and establishment of hairy roots cultures

For genetic transformation, we used the leaves of *F. multiflora* as explants and precultured them on MS medium for 2 d. For inoculation, the control/OE/RNAi groups of C58C1 were cultured in the dark in a liquid TY medium supplemented with 100 mg/L rifampicin and 50 mg/L spectinomycin (spectinomycin was not required for the control group) at 200 rpm for 15 h at 28°C. A 500 μL aliquot of the cultures was then sub-cultured in a 50 mL liquid TY medium at 200 rpm and 28°C until the OD_600_ ≈ 0.6-0.8, after which the subculture could be used for the genetic transformation. We then immersed the explants in different groups of C58C1 under shaking at 130 rpm for 25 min. Then the explants were picked out and patted dry on sterile filter paper, and co-cultured on solid MS medium in the dark at 25°C for 2 d. Next, the explants were transferred to solid MS medium supplemented with 400 mg/L Cefotaxime sodium (Cef) for sterilization. The hairy roots grew from the leaf margins and reached 3-5 cm in length in two weeks at which time the OE and RNAi group hairy roots needed to be transferred onto the solid MS medium containing 400 mg/L Cef and 50 mg/L Kanamycin for selective culture. During this period, we gradually reduced the concentration of Cef (400 mg/L, 200 mg/L, 100 mg/L and 0 mg/L) every 3 d to ensure that the selected hairy roots could be continually grown. The Selected hairy root lines that remained strong after the selective culture were transferred into liquid MS medium (pH= 5.7; antibiotic free) in the dark at 25°C and 130 rpm for 3 weeks and then sub-cultured in new liquid MS medium two times repeatedly to obtain putatively transformed hairy root lines with stable growth.

### Identification and quantitative reverse transcription PCR analysis of transgenic hairy roots

Different groups of the putatively transformed hairy roots were twice weighed out to 0.1000 g each and ground into powder with liquid nitrogen for PCR and RT-qPCR analysis. TaKaRa MiniBEST Plant Genomic DNA Extraction Kit (TaKaRa, Japan) was used for the DNA extraction of the hairy roots for molecular biological detection. The PCR primers are shown in Table S6. We performed PCR analysis using the 2 x *Accurate Taq* Master Mix (dye plus; Accurate Biology, China). Additionally, the pK7WG2D and pK7GWIWG2D (II) plant expression plasmid both carry the *enhanced green fluorescent protein* (*eGFP*) gene, which can induce the transgenic organs to produce green fluorescence, meaning that the expression of eGFP in the transgenic hairy roots could be detected using the OLYMPUS BX53 fluorescent microscope (OLYMPUS, Japan).

We conducted RT-qPCR analysis of *FmUGT1* and *FmUGT2* genes using a CFX96 Touch (BioRad, Hercules, CA, USA) and extracted the total RNA of the hairy roots using an RNAprep Pure Plant Kit (Tiangen, China). After this, an Evo M-MLV RT Kit with gDNA Clean for qPCR II (Accurate Biology, China) was used to reverse transcribe the total RNA and erase the genomic DNA. The *F. multiflora PPAP* gene was then used as the internal control to normalize expression levels of the target genes among different groups of samples, and the primers are shown in Table S6. Next, RT-qPCR was performed using a SYBR^®^ Green Premix Pro *Taq* HS qPCR Kit II (Accurate Biology, China). The RT-qPCR programs were as follows: pre-denaturation at 95°C for 30 s, 40 cycles of denaturation at 95°C for 5 s, and annealing/extension at 60°C for 30 s with fluorescence reading. Finally, we calculated three biological replicates and three technical replicates using the 2^-ΔΔCt^ method (Livak and Schmittgen, 2001).

### Analysis of THSG content by HPLC- Photodiode Array

In order to analyze THSG content, different groups of transgenic hairy roots were sub-cultured in a new liquid MS medium (pH=5.7) in the dark at 25°C and 130 rpm for 45 d prior to compounds contents assay. The stilbenes were then extracted as described by Zhu et al. (2013). First, the samples (0.2000 g each) were finely ground under liquid nitrogen and dried at 45°C. They were then soaked with 25 mL of diluted ethanol (52.9:47.1, V: V) for 12 h, and were resuspended every 2 h during this period. These extracts were centrifuged at 5,500 rpm for 20 min to remove impurities, and the supernatant was filtered through a 0.45 μm nylon filter (Jinlong, China) as test solution. Analysis of the test solution was performed on a Waters 1525 HPLC system equipped with a PDA detector, and separation was achieved using a Phenomenex 00G-4252-E0 (250 mm × 4.6 mm I.D., 0.45 μm) with the gradient program set to: acetonitrile (A): water (B) (25: 75, V: V) for 20 min. The flow rate was 1.0 mL/min, and the injection volume was 20 μL, with a detection wavelength of 320 nm. Finally, we dissolved THSG standard in diluted ethanol and diluted it to 8 gradient concentrations (700.00, 350.00, 175.00, 87.50, 43.75, 21.88, 10.94 and 5.47 μg/mL). The standard curve was then plotted with the concentrations and peak areas as the abscissa and ordinate, respectively.

### Analysis of AQs content by HPLC-PDA

We extracted the AQs compounds using the method described by Thiruvengadam et al. (2014) with some modifications. As with the above THSG analysis, the samples (0.2000 g each) were finely ground under liquid nitrogen and dried at 45°C. They were then extracted with methanol (5 mL×6) under 20 min sonication to ensure the complete extraction of AQs. These extracts were centrifuged at 5,500 rpm for 20 min to remove impurities and filtered by 0.45 μm nylon filter (Jinlong, China) as test solution. The analysis of the test solution was performed on a Waters 1525 HPLC system equipped with a PDA detector, and separation was achieved using a Phenomenex 00G-4252-E0 (250 mm × 4.6 mm I.D., 0.45 μm) with the gradient program set to: methanol (A): 0.1% phosphoric aqueous acid (B) (20: 80, V: V) for 20 min. The injection volume was 20 μL and the flow rate was 1.0 mL/min, with a detection wavelength of 254 nm. We purchased emodin and physcion standard from Macklin Biochemical Co., Ltd (China), which we dissolved in methanol until their mother liquor concentrations were 230 and 116 μg/mL, respectively, and diluted them to different concentration. The ranges of concentration for the emodin standard were 230.00, 115.00, 57.50, 28.75, 14.38, 7.19 and 3.59 μg/mL, and for the physcion standard were 116.00, 58.00, 29.00, 14.50, 7.25 and 3.63 μg/mL. Finally, we plotted the standard curves with the concentrations and peak areas as the abscissa and ordinate, respectively.

### Statistical analysis

The data from our LC-MS/MS analysis were completed using SCIEX OS software (Version 1.4; AB SCIEX, Framingham, MA, USA). Comparisons of the levels of *FmUGT1* and *FmUGT2* gene expression, THSG, and AQs contents of different groups of transgenic hairy roots were analyzed using one-way ANOVA from three independent biological replicates with three technical replicates each. All data are shown as mean ± *SD*. We used SPSS (Version 26.0; SPSS Inc., Chicago, IL, USA) for data analysis and statistical calculations, and generated graphs with GraphPad Prism (version 8.0; GraphPad Software, San Diego, CA, USA).

## Results

### Full-length sequence analysis and recombinant protein purification of *FmUGT1* and *FmUGT2*

We predicted the physicochemical properties of the *FmUGT1* and *FmUGT2* (Table S7) using ExPASY ProtParam, and both of them were predicted to be unstable hydrophilic proteins. The SignalP 5.0 predicted that there were no transmembrane region for *FmUGT1* and *FmUGT2*, and the Cell PLOC 2.0 subcellular localization predicted that *FmUGT1* was located in the cell membrane and that *FmUGT2* was located in the chloroplast. The predicted secondary structures of *FmUGT1* and *FmUGT2* are shown in Table S8. The three-dimensional models of *FmUGT1* and *FmUGT2* were constructed by SWISS-MODEL, using 6l90.1.A (Li et al., 2020) and 2acw.1.A (Shao et al., 2005) as templates, respectively, and their homologies were 27.29% and 46.99% for *FmUGT1* and *FmUGT2*, respectively (**Figure S1**).

**Figure 2:**
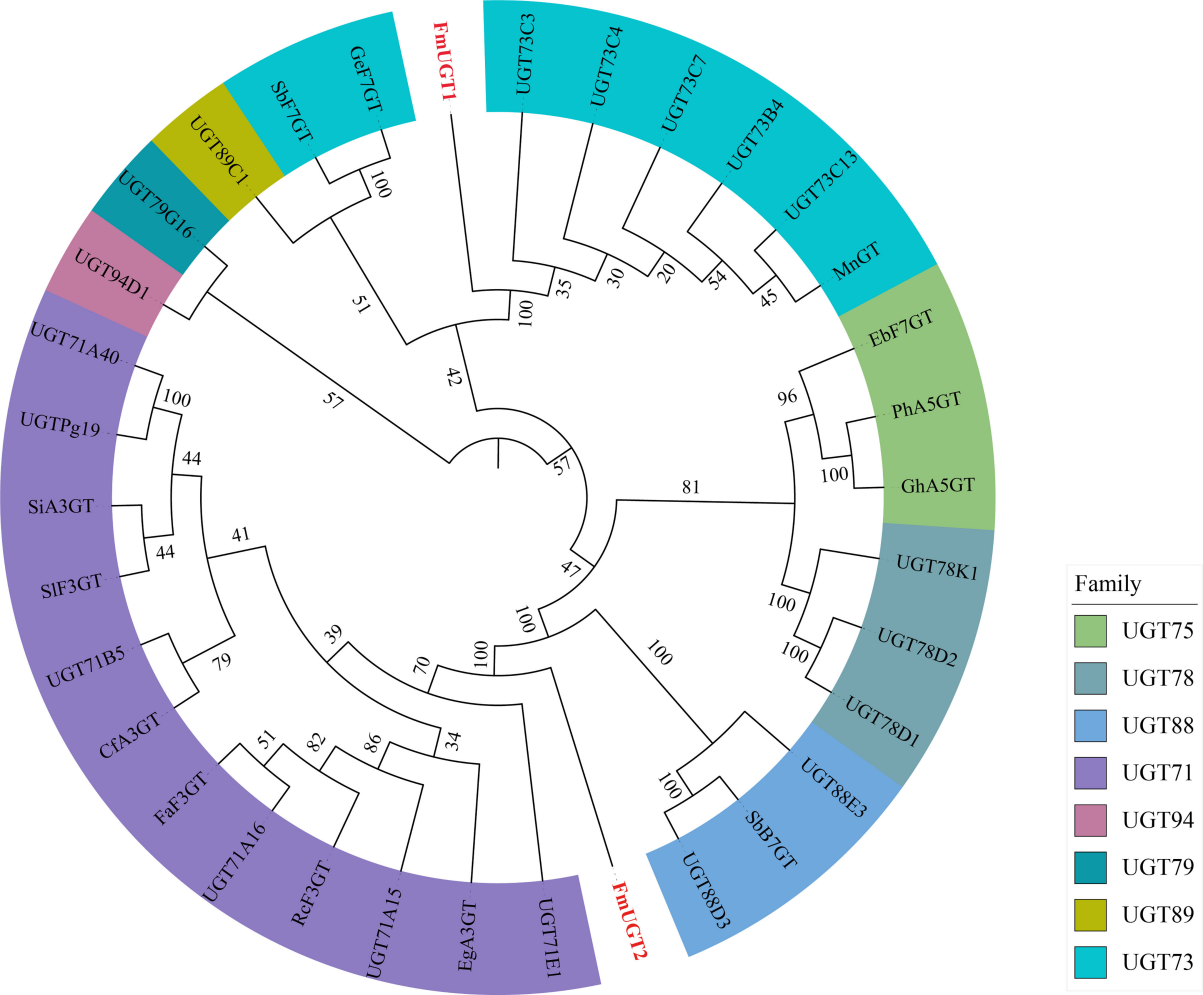
Phylogenetic analysis of *FmUGT1* and *FmUGT2* with other UGTs. FmUGTs from this study were colored in red. The GenBank accession numbers of UGT proteins in the tree are in **Supplementary Table 7**.

Multiple sequence alignment result displayed the similarity of amino acid sequence among *FmUGT1*, *FmUGT2* as well as the homologs in other species; specific functional domain Plant Secondary Product Glycosyltransferase (PSPG) boxes containing highly conserved sequences “HCGWNS” were also indicated (**Figure S2**). Additionally, we selected GTs with different functions from other plants for phylogenetic tree analysis, and the results showed that *FmUGT1* was most closely related to UGT95D1 from *Fagopyrum tataricum* and the *FmUGT2* was close to the UGT71 family (**Figure 2**).

Next in our analysis, results from SDS-PAGE (**Figure S3**) show that the molecular weights of *FmUGT1* and *FmUGT2* were both consistent with their theoretical values (Table S7). We thus conclude that *FmUGT1* and *FmUGT2* were indeed successfully induced in this study by the process described above. Using Ni-NTA to purify the soluble proteins induced from *FmUGT1* and *FmUGT2*, we successfully eluted the purified *FmUGT1* and *FmUGT2* using Ni-NTA columns with the 0.05 M Tris-HCl buffer (pH= 7.4) containing 150 mM and 200 mM imidazole, respectively, and the SDS-PAGE results show that the purified protein bands were consistent with those of the induced proteins (**Figure S3**).

### Assaying catalytic parameters and glycosylation activity of *FmUGT1* and *FmUGT2*

**Figure 3:**
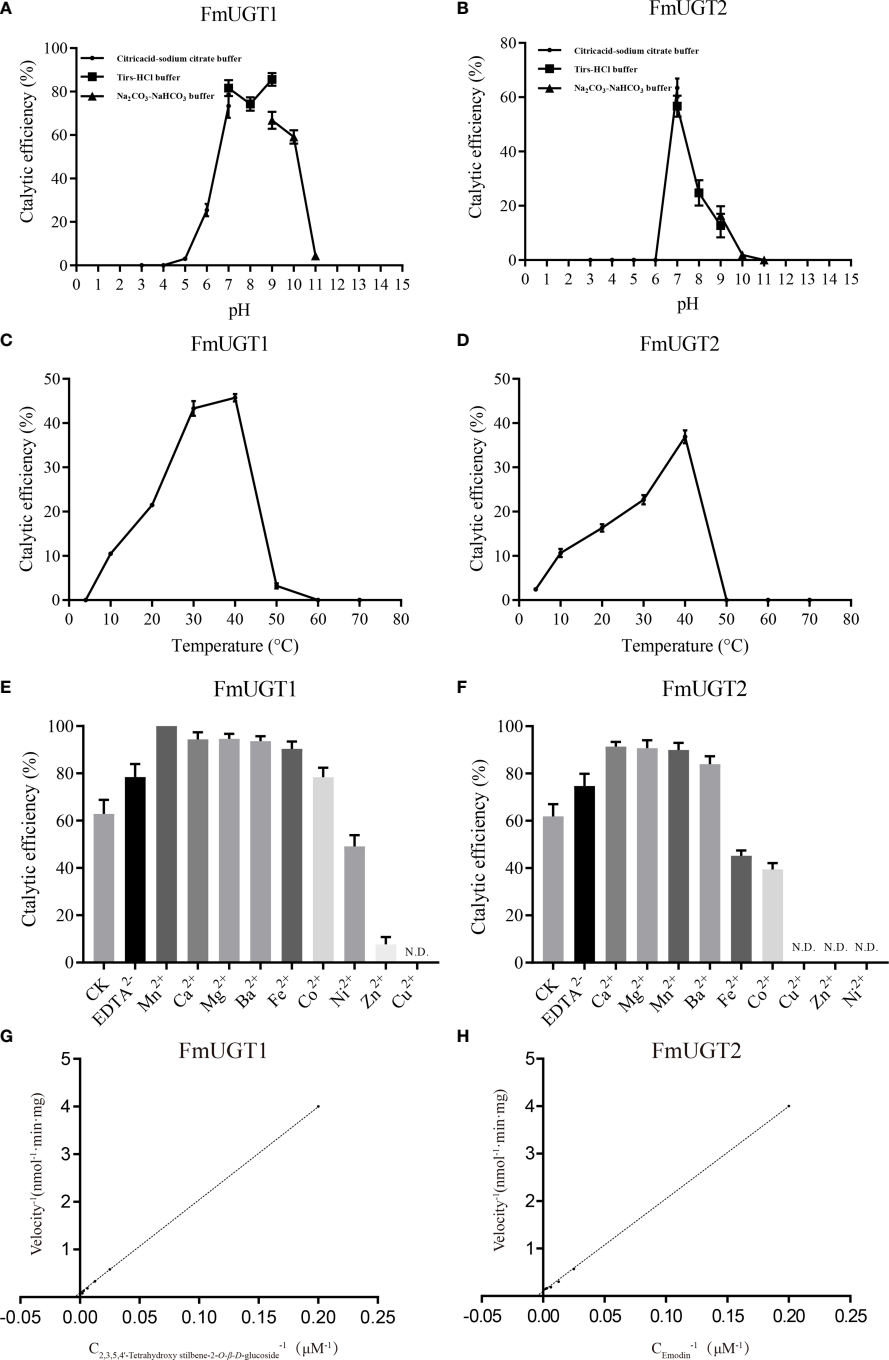
Biochemical properties of recombinant protein FmUGT1 and FmUGT2. **(A)** Effect of pH on the enzyme activity of FmUGT1. **(B)** Effect of pH on the enzyme activity of FmUGT2. The points represent Citric acid-sodium citrate Buffer (pH3.0-7.0), the squares represent Tris-HCl buffer (pH7.0-9.0), the triangle represent Na_2_CO_3_-NaHCO_3_ buffer (pH9.0-11.0). **(C)** Effect of temperature on the enzyme activity of FmUGT1. **(D)** Effect of temperature on the enzyme activity of FmUGT2. **(E)** Effect of metal cations on the enzyme activity of FmUGT1. **(F)** Effect of metal cations on the enzyme activity of FmUGT2. **(G)** The Lineweaver-Burk plot of FmUGT1. **(H)** The Lineweaver-Burk plot of FmUGT2. Values of the relative activities are mean ± *SD* (n=3).

We found that FmUGT1 and FmUGT2 exhibits its maximum activity at pH 9.0, 40°C, and at pH 7.0, 40°C, respectively (**Figures 3A-D**). The effect of metal cations showed that FmUGT1 and FmUGT2 are non-cation-dependent protein, which are significantly inhibited by Zn^2+^, Cu^2+^ and Ni^2+^ (**Figures 3E, F**). The Lineweaver-Burk plot (**Figures 3G, H**) shown that the *V_max_, K_m_, K_cat_* and *K_cat_*/*K_m_* value of FmUGT1 and FmUGT2 were 12.3 nM^-1^·min^-1^·mg^-1^, 236 μM, 0.1975 s^-1^ and 0.8369 mM^-1^·s^-1^; 9.4 nM^-1^·min^-1^·mg^-1^, 185 μM, 0.1413 s^-1^ and 0.7637 mM^-1^·s^-1^ respectively.

**Figure 4:**
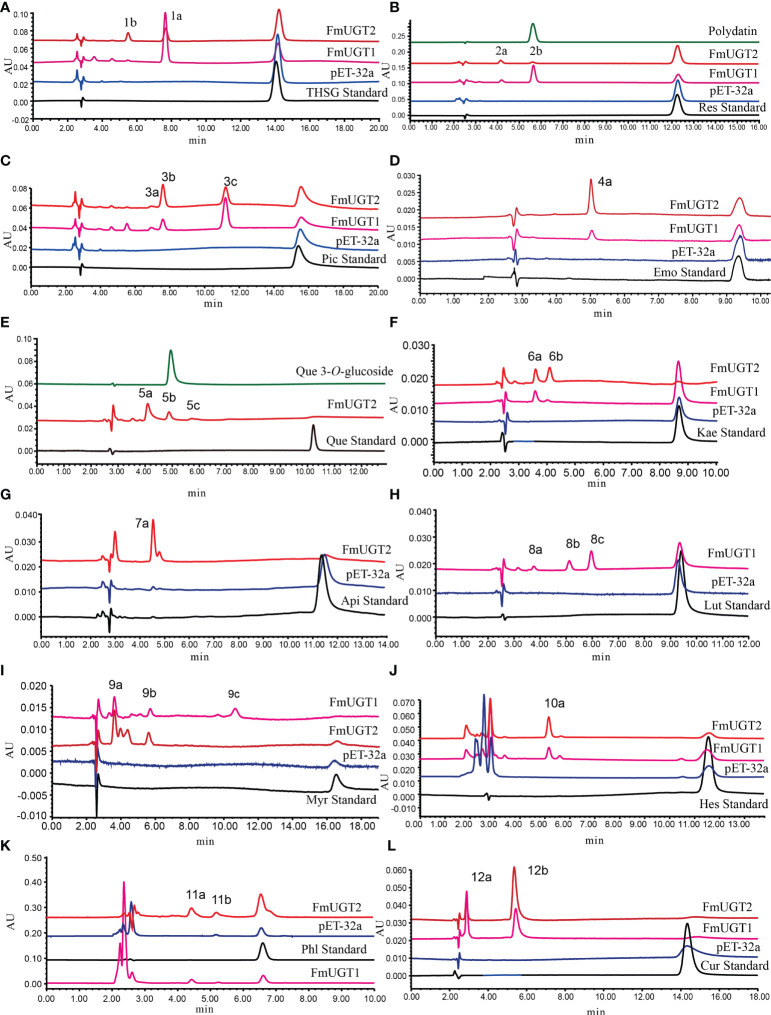
Functional assays of *FmUGT1* and *FmUGT2* using UDP-glucose as the donor and a range of substrates. The reaction mixtures were incubated at 30°C for 12 h. **(A)** THSG; **(B)** Resveratrol (Res); **(C)** Piceatannol (Pic); **(D)** Emodin (Emo); **(E)** Quercetin (Que); **(F)** Kaempferol (Kae); **(G)** Apigenin (Api); **(H)** Luteolin (Lut); **(I)** Myricetin (Myr); **(J)** Hesperetin (Hes); **(K)** Phloretin (Phl); **(L)** Curcumin (Cur).

From our *in vitro* enzyme assays, HPLC analysis showed that *FmUGT1* could convert substrates 1-6, 8-12 to produce new peaks (**Figure 4**), and the conversion rate of substrates 1, 3, 5, 9, 10, and 12 were all higher than 50%. In addition, *FmUGT2* could convert substrates 1-12 to produce new peaks (**Figure 4**), and the conversion rate of most of these were also higher than 50%, especially for substrates 5, 6, 8, and 9, whose conversion rates reached nearly 100% (**Figure 1**).

### Structural identification of *FmUGT1* and *FmUGT2* glycosylation products

To determine the glycosylation functions of *FmUGT1* and *FmUGT2*, the catalytic products 2b and 5b were identified using polydatin (resveratrol 3-*O*-glucoside) and quercetin 3-*O*-glucoside standard by comparing their retention time in the HPLC spectrum (**Figures 4B, E**). And then 11 putative glycosylated products were also isolated and concentrated from the preparative-scale reactions, and their structures were identified using LC-MS/MS, ^1^H-NMR, and ^13^C-NMR spectroscopic analysis compared to already-reported data. The structures of the glycosylated products are shown in **Figure 1**, the LC-MS/MS analysis results are shown in **Figures S5-15**, and the ^1^H-NMR and ^13^C-NMR results are listed below and also in **Figures S16-37**.

### Identification of the transgenic hairy roots

**Figure 5:**
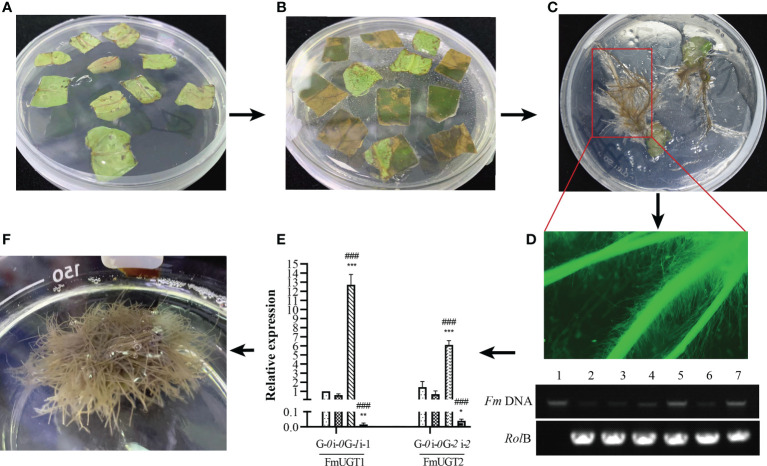
The process of transgenic hairy roots construction. **(A)** Preculture of the detached *F multiflora* leaves. **(B)** Continued culture of the detached **(F)** *multiflora* leaves after C58C1 infection. **(C)** Hairy roots grew at the leaf margins. **(D)** Identification of the hairy roots. Observation of eGFP protein expression through fluorescence microscope (top); PCR analysis of the *RolB* gene (bottom). **(E)** RT-qPCR analysis of *FmUGT1* and *FmUGT2* in different groups of transgenic hairy roots. G-*0*: pK7WG2D-*0*; i-*0*: pK7GWIWG2D-*0*; G-*1*: pK7WG2D-*FmUGT1*; i-*1*: pK7GWIWG2D-*FmUGT1i*; G-*2*: pK7WG2D-*FmUGT2*; i-*2*: pK7GWIWG2D-*FmUGT2i*. **(F)** The identified hairy roots were cultured in MS liquid culture medium. * Indicates a significant difference between the control groups and OE/RNAi groups at *P* < 0.05; ** indicates a significant difference at *P* < 0.01; and *** indicates a significant difference at *P* < 0.001. ### Indicates a significant difference between the OE groups and RNAi groups at *P* < 0.001. Every group contained three biological replicates with three technical replicates each. The vertical bar represents mean ± *SD* (n = 3).

Hairy roots were induced from leaf margins after preculture, transformation, and co-culture (**Figures 5A–C**). Their *RolB* gene was then cloned using PCR, and green fluorescence was observed using a fluorescence microscope (**Figure 5D**). The RT-qPCR results showed that the relative expression of *FmUGT1* and *FmUGT2* in the OE groups (pK7WG2D) were both upregulated (*P*<0.001) by 12.61-fold and 4.15-fold compared to the pK7WG2D-*0*, respectively and that the relative expression of *FmUGT1* and *FmUGT2* in the RNAi groups (pK7GWIWG2D (II)) were 36.25-fold (*P*<0.01) and 17.33-fold (*P*<0.05) lower than in pK7GWIWG2D-*0*, respectively. Moreover, compared to the OE groups, the relative expression levels of *FmUGT1* and *FmUGT2* were 0.13% and 0.62% of the level in the RNAi groups (*P*<0.001), respectively (**Figure 5E**). These results indicate that the target genes were ideally overexpressed or interfered in corresponding groups, and we thus conclude that we indeed obtained transgenic hairy roots in this research (**Figure 5F**).

### Determination of THSG and AQs contents in transgenic hairy roots

**Figure 6:**
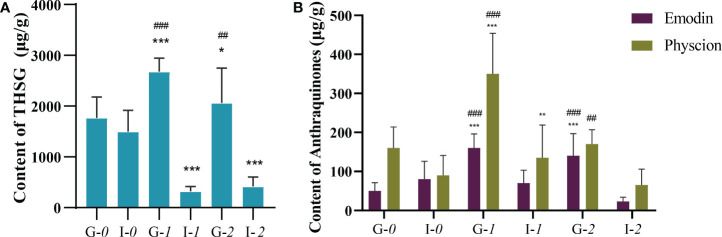
Determination of THSG and AQs contents in hairy roots. **(A)** The contents of THSG in each group. **(B)** The contents of AQs in each group. G-*0*: pK7WG2D-*0*; i-*0*: pK7GWIWG2D-*0*; G-*1*: pK7WG2D-*FmUGT1*; i-*1*: pK7GWIWG2D-*FmUGT1i*; G-*2*: pK7WG2D-*FmUGT2*; i-*2*: pK7GWIWG2D-*FmUGT2i*. * Indicates a significant difference between the control groups and OE/RNAi groups at P < 0.05, ** Indicates a significant difference between the control groups and OE/RNAi groups at *P* < 0.01, and *** indicates a significant difference at *P*<0.001. ### Indicates a significant difference at *P* < 0.001 between the OE groups and RNAi groups. Every group contained three biological replicates with three technical replicates each. The vertical bar represents mean ± *SD* (n = 3).

The results of the assay of the content of stilbene in the hairy roots of each group are shown in **Figure 6A**. We detected the THSG content in the pK7WG2D-*FmUGT1* hairy roots to be 2679.44 μg/g, which was 51.44% (*P*<0.001) and 7.37-fold higher (*P*<0.001) than pK7WG2D-*0* and pK7GWIWG2D-*FmUGT1i*, respectively. In addition, the content of THSG in the pK7WG2D-*FmUGT2* hairy roots was 2027.37 μg/g, which was 16.79% (*P*<0.05) and 3.88-fold higher (*P*<0.01) than in pK7WG2D-*0* and pK7GWIWG2D-*FmUGT2i*, respectively. In contrast, the THSG content in pK7GWIWG2D-*FmUGT1i* and pK7GWIWG2D-*FmUGT2i* was 78.61% and 72.26% lower, respectively, than in pK7GWIWG2D-*0* (*P*< 0.001).

Results for the AQs contents in the hairy roots of each group are shown in **Figure 6B**. The quantitative detection of emodin and physcion, shows that emodin content in pK7WG2D-*FmUGT1* was 164.15 μg/g, which was higher (*P*<0.001) by 2.00-fold and 1.26-fold compared to pK7WG2D-*0* and pK7GWIWG2D-*FmUGT1i*, respectively. For pK7WG2D-*FmUGT2*, the emodin content was 147.62 μg/g, which was 1.70-fold and 5.21-fold higher (*P*< 0.001) than in pK7WG2D-*0* and pK7GWIWG2D-*FmUGT2*i respectively. Physcion content in the pK7WG2D-*FmUGT1* hairy roots was 337.36 μg/g, which was higher (*P*< 0.001) by 1.07-fold and 1.49-fold compared to pK7WG2D-*0* and pK7GWIWG2D-*FmUGT1i*, respectively, and for the pK7WG2D-*FmUGT2* hairy roots the concentration was 170.75 μg/g, which was 1.61-fold higher (*P*<0.05) than in pK7GWIWG2D-*FmUGT2i*. However, we found no statistically significant difference between pK7WG2D-*FmUGT1* and pK7WG2D-*0.*

## Disscussion

*FmUGT1* and *FmUGT2* showed substrate promiscuity through *in vitro* catalytic reaction and successfully catalyzed the glycosylation of 12 substrates with different structures. Through structural analysis of the glycosylation products, we found that *FmUGT1* and *FmUGT2* could catalyze the 3-*O*-glycosylation of THSG and resveratrol to form polygonimitin C and polydatin, as well as could also cause the 3’-*O*-glycosylation of piceatannol to form piceatannol 3’-*O*-glucoside. Unfortunately, when we tried to prepare the 2,3,5,4’- tetrahydroxystilbene compound from THSG using acidic hydrolysis method, it was found to be severely degraded within 24 h, which was also demonstrated by Ren et al. (2011). Therefore, we failed to prepare the 2,3,5,4’- tetrahydroxystilbene for the *in vitro* glycosylation catalysis studies. When using emodin as the substrate, *FmUGT1* and *FmUGT2* both tended to transfer sugars from glycoside scaffolds to an 8-*O* position at the aglycon to form emodin 8-*O*-glucoside. According to the glycosylation sites of compound 5b, 6a, and 9a, *FmUGT1* and *FmUGT2* most frequently attached glycosyl at 3-*O*. However, in the absence of the 3-OH, *FmUGT1* and *FmUGT2* tended to form 7-*O*-glycosylation of flavonoids (compound 7a, 8a, and 10a).

Interestingly, phloretin, a dihydrochalcone, is structurally similar to apigenin, and *FmUGT1* and *FmUGT2* were both more prone to attaching glycosyl at the 4’-*O* position, which is similar to the 7-*O* position at the A-ring of flavonoids, suggesting that *FmUGT1* and *FmUGT2* might have stereoselectivity during glycosylation. Additionally, *FmUGT1* could catalyze 5’-*O*-glycosylation of myricetin to form compound 9c, and *FmUGT1* and *FmUGT2* could both catalyze the 4’-*O* of curcumin to form compound 12a, which helped improve the solubility of curcumin. These results indicate that *FmUGT1* and *FmUGT2* demonstrated substrate promiscuity and possibly stereoselectivity, which may further supplement the glycosylation tool enzyme library and provide a new method for the synthesis of glycosides.

To study the role of *FmUGT1* and *FmUGT2* in the synthesis of secondary metabolites of *F. multiflora* further, we constructed pK7WG2D-*FmUGT1*, pK7GWIWG2D-*FmUGT1i*, pK7WG2D-*FmUGT2*, and pK7GWIWG2D-*FmUGT2i* recombinant plant expression vectors for genetic modification. Molecular biology detection and RT-qPCR detection from these efforts showed that *FmUGT1* and *FmUGT2* were successfully overexpressed or interfered with in corresponding groups of hairy roots. Moreover, our RT-qPCR results displayed that the relative expressions of *FmUGT1* and *FmUGT2* in the RNAi group were significantly downregulated compared with the control groups and that their THSG contents were correspondingly decreased. However, the results of OE-*FmUGT1* and OE-*FmUGT2* were quite opposite to those of the RNAi groups. In the former groups the relative expressions were upregulated significantly compared to those of the control groups, and their THSG contents were also significantly increased, indicating that *FmUGT1* and *FmUGT2* play a key role in the THSG biosynthesis pathway in *F. multiflora*.

Similarly, the contents of emodin and physcion in OE-*FmUGT1* and OE-*FmUGT2* were significantly higher than those in the control and RNAi groups, but after interfering with the expression of *FmUGT1* and *FmUGT2*, the contents of emodin and physcion were not significantly different from those in the control groups, showing that although promoting the transcription of *FmUGT1* and *FmUGT2* could help enhance the production of AQs in the hairy roots of *F. multiflora*, *FmUGT1* and *FmUGT2* were not key members of the AQs biosynthesis pathway. Additionally, we also tried to detect the flavonoid content of different groups of hairy roots using HPLC-PDA, but could not find any relevant flavonoids.

## Conclusion

In summary, we found that *FmUGT1* and *FmUGT2* showed substrate promiscuity and could catalyze 12 substrates of stilbenes, AQs, flavonoids, phloretin, and curcumin to produce corresponding glucosides. Our analysis of the *in vivo* function of *FmUGT1* and *FmUGT2* further showed that *FmUGT1* and *FmUGT2* could efficiently catalyze the glycosylation of THSG and promote the production of AQs in *F. multiflora* transgenic hairy roots. Taken together, the above results will provide a crucial foundation to help clarify the molecular pathway of THSG biosynthesis.

## Data availability statement

The datasets presented in this study can be found in online repositories. The names of the repositories and accession numbers can be found below: https://www.ncbi.nlm.nih.gov/nuccore/ON262204.1; https://www.ncbi.nlm.nih.gov/nuccore/ON262205.1.

## Author contributions

LZ, and QY conceived and designed the experiments. QC, CL and LL performed the experiments and drafted the manuscript. QC, CL, YG, XC and BL analyzed the data. All authors contributed to the discussion and revised and approved the final manuscript. All authors contributed to the article and approved the submitted version.

## Funding

This work was supported by National Key Research and Development Program (2017YFC1700704), Special Fund for the Protection of Lingnan Chinese Medicinal Materials in Guangdong Province in 2017 (Yue cai she [2017] No. 60), and Guangdong Provincial Youth Innovation Talents Program (2018KQNCX133).

## Conflict of interest

The authors declare that the research was conducted in the absence of any commercial or financial relationships that could be construed as a potential conflict of interest.

## Publisher’s note

All claims expressed in this article are solely those of the authors and do not necessarily represent those of their affiliated organizations, or those of the publisher, the editors and the reviewers. Any product that may be evaluated in this article, or claim that may be made by its manufacturer, is not guaranteed or endorsed by the publisher.
