# Supplementary material for: Molecular identification and functional characterization of two glycosyltransferases genes from Fallopia multiflora
Source: Front Plant Sci. 2022 Dec 6;13:1017122. doi: 10.3389/fpls.2022.1017122 (PMC9765892; doi:10.3389/fpls.2022.1017122)
Supplement: Supplementary file 1 [file DataSheet_1.pdf]

Supplementary Material

Supplementary Tables

Supplementary TABLE 1. Characteristic primer pairs for PCR and RT-qPCR amplification of *FmUGT1* and *FmUGT2*

Primers name	Sequence (From 5' to 3')	Length（bp）
pET-32a-*FmUGT1*-F	GCCATGGCTGATATCGGAATGGCGGTCGCCGGCGAAAT	1635
pET-32a-*FmUGT1*-R	GAGCTCGAATTCGGATCACTCTGTCTTCGCACCCACGAAA	
pET-32a-*FmUGT2*-F	GCCATGGCTGATATCGGAATGACCATTTCAAAGACGGC	1482
pET-32a-*FmUGT2*-R	GAGCTCGAATTCGGATCAAGGGAGATTCTTAAACACAA	

Supplementary TABLE 2. HPLC methods for detection of glycosylation products catalyzed by FmUGT1 and FmUGT2.

Method	Solvent A	Solvent B	Gradient	Substrates
A	CH_3OH	H_2O	35% A, 65% B, 20 min	1, 3
B	CH_3OH	H_2O	40 % A, 60% B, 16 min	2, 6
C	CH_3OH	H_2O	80 % A, 20% B, 15 min	4
D	CH_3OH	H_2O	55 % A, 45% B, 15 min	5, 7, 8, 9, 10
E	CH_3OH	H_2O	60 % A, 40% B, 15 min	11
F	CH_3OH	H_2O	65 % A, 35% B, 20 min	12

Supplementary TABLE 3. UPLC methods for detection of glycosylation products catalyzed by FmUGT1 and FmUGT2.

Method	Solvent A	Solvent B	Gradient	Substrates
A	CH3CN	H_2O	20-30% A, 3 min; 30-20% A, 0.5 min; 20% A, 1.5 min	1-3
B	CH3CN	H_2O	20-90% A, 4.5 min; 90-20% A, 0.5 min; 20% A, 2 min	4
C	CH3CN	H_2O	5-25% A, 1 min; 25-40% A, 2.5 min; 40-60% A, 1 min; 60-5% A, 0.5 min; 5% A, 2 min	5-12

Supplementary TABLE 4. Semi-HPLC methods for detection of glycosylation products catalyzed by FmUGT1 and FmUGT2.

Method	Solvent A	Solvent B	Gradient	Substrates
A	CH_3OH	H_2O	28% A, 72% B, 35 min	1, 3
B	CH_3OH	H_2O	33 % A, 67% B, 30 min	2, 6
C	CH_3OH	H_2O	75 % A, 25% B, 25 min	4
D	CH_3OH	H_2O	45 % A, 55% B, 35 min	5, 7, 8, 9, 10
E	CH_3OH	H_2O	52 % A, 48% B, 30 min	11
F	CH_3OH	H_2O	56 % A, 44% B, 30 min	12

Supplementary TABLE 5. Primers used for plant expression vector construction.

Primers name	Primers sequence (5’→3’)	Length（bp）
pDONR-*FmUGT1-F*	GGGGACAAGTTTGTACAAAAAAGCAGGCTCCATGGCGGTCGCCGGCGAA	1663
pDONR-*FmUGT1-R*	GGGGACCACTTTGTACAAGAAAGCTGGGTGTCACTCTGTCTTCGCACC	
pDONR-*FmUGT2-F*	GGGGACAAGTTTGTACAAAAAAGCAGGCTCCATGACCATTTCAAAGACG	1510
pDONR-*FmUGT2-R*	GGGGACCACTTTGTACAAGAAAGCTGGGTGTCAAGGGAGATTCTTAAA	
pDONR-*FmUGT1i-F*	GGGGACAAGTTTGTACAAAAAAGCAGGCTCCCAGGCGGGTTCTTGACTCAT	305
pDONR-*FmUGT1i-R*	GGGGACCACTTTGTACAAGAAAGCTGGGTGGCCATAGCCCTTGTTTTCGC	
pDONR-*FmUGT2i-F*	GGGGACAAGTTTGTACAAAAAAGCAGGCTCCACCTTCAACGACACGCAGAT	308
pDONR-*FmUGT2i-R*	GGGGACCACTTTGTACAAGAAAGCTGGGTGATCCACAGTGCGACACGAAT	

Supplementary TABLE 6. PCR and RT-qPCR primers used for transgenic hairy root identification.

Purpose	Primers name	Primers sequence (5'→3')	Length（bp）
PCR	*eGFP*-F	CTCGTTGGGGTCTTTGCTC	567
PCR	*eGFP*-R	CACAAGTTCAGCGTGTCCG	
PCR	*RolB*-F	GCTCTTGCAGTGCTAGATTT	432
PCR	*RolB*-R	GAAGGTGCAAGCTACCTCTC	
PCR	pK7WG2D-F	GGTGTTGTTTTGTTTGTTGTTTGTTGT	/
PCR	*FmUGT11*-R	TCACTCTGTCTTCGCACCCA	1770
PCR	*FmUGT36*-R	TCAAGGGAGATTCTTAAACAC	1632
PCR	pK7GWIWG2D-F1	CTTATATGCTCAACACATGAGCGAAA	/
PCR	*FmUGT11i*-R	GCCATAGCCCTTGTTTTCGC	523
PCR	*FmUGT36i*-R	ATCCACAGTGCGACACGAAT	526
PCR	*antisense*-pK7GWIWG2D-F	GCCGTAAGAAGAGGCAAGAGTATGAAA	/
PCR	*antisense*-*FmUGT11i*-R	ATGAGTCAAGAACCCGCCTG	448
PCR	*antisense*-*FmUGT36i*-R	ATCTGCGTGTCGTTGAAGGT	451
RT-qPCR	*PP2A*-F	CTCGTTGGGGTCTTTGCTC	101
RT-qPCR	*PP2A*-R	CACAAGTTCAGCGTGTCCG	
RT-qPCR	*FmUGT1*-F	CAGGCGGGTTCTTGACTCAT	102
RT-qPCR	*FmUGT1*-R	TGTAGAACTGGTCGCCCCTA	
RT-qPCR	*FmUGT2*-F	CATGTCGCAGCAAGACATCG	115
RT-qPCR	*FmUGT2*-R	TGGATCTTTCCCGGAGGACT	

Supplementary TABLE 7. Physicochemical property properties of *FmUGT1* and *FmUGT2*.

Protein name	Amino acid	Protein formula	Molecular weight	PI	Instability index (II)	GRAVY	Subcellular localization
FmUGT1	533	$C_{2554}H_{3926}N_{688}O_{742}S_{24}$	56.91	6.11	43.99	-0.303	Cell membrane
FmUGT2	482	$C_{2403}H_{3791}N_{635}O_{700}S_{15}$	53.25	5.38	48.05	-0.069	Chloroplast

Supplementary TABLE 8. Predicted secondary structure of *FmUGT1* and *FmUGT2*.

Protein name	Random coil	α-Helices	Extended strand	β-Turn
FmUGT1	44.09 %	37.90 %	11.63 %	6.38 %
FmUGT2	39.00 %	42.32 %	13.49 %	5.19 %

Supplementary TABLE 9. GenBank accession numbers of UGT proteins in Figure 3.

Gene	Accession number	Species
UGT71B5	GFZ12326.1	*Actinidia rufa*
UGT73B4	GFZ16622.1	*A. rufa*
UGT78D1	NP_564357.1	*Arabidopsis thaliana*
UGT78D2	NP_197207.1	*A. thaliana*
UGT89C1	Q9LNE6.1	*A. thaliana*
UGT71E1	PWA74166.1	*Artemisia annua*
UGT88D3	Q33DV3.1	*Antirrhinum majus*
UGT73C3	XP_016201209.1	*Arachis ipaensis*
CfA3GT	AUM57507.1	*Camellia fraterna*
EbF7GT	ANC70234.1	*Erigeron breviscapus*
EgA3GT	XP_010045032.2	*Eucalyptus grandis*
FaF3GT	Q66PF3.1	*Fragaria x ananassa*
GhA5GT	Q9ZR25.1	*Glandularia x hybrida*
UGT78K1	ADC96620.1	*Glycine max*
UGT88E3	NP_001235161.1	*G. max*
GeF7GT	BAC78438.1	*Glycyrrhiza echinata*
UGT79G16	Q53UH5.1	*Ipomoea purpurea*
UGT71A40	QEA68966.1	*Panax ginseng*
UGTPg19	AKA44592.1	*P. ginseng*
PhA5GT	BAA89009.1	*Petunia x hybrida*
UGT71A16	XP_007217386.1	*Prunus persica*
UGT73C4	XP_002512998.1	*Ricinus communis*
UGT73C7	XP_024191911.1	*Rosa chinensis*
RcF3GT	XP_024168185.1	*R. chinensis*

SbF7GT	BAA83484.1	*Scutellaria baicalensis*
SbB7GT	Q76MR7.1	*S. baicalensis*
UGT94D1	BAF99027.1	*Sesamum indicum*
SiA3GT	XP_011100453.1	*S. indicum*
SlF3GT	XP_004243651.1	*Solanum lycopersicum*
UGT71A15	XP_010093123.1	*M. notabilis*
MnGT	XP_010110434.1	*M. notabilis*
UGT73C13	XP_021607184.1	*M. notabilis*

Supplementary Figures

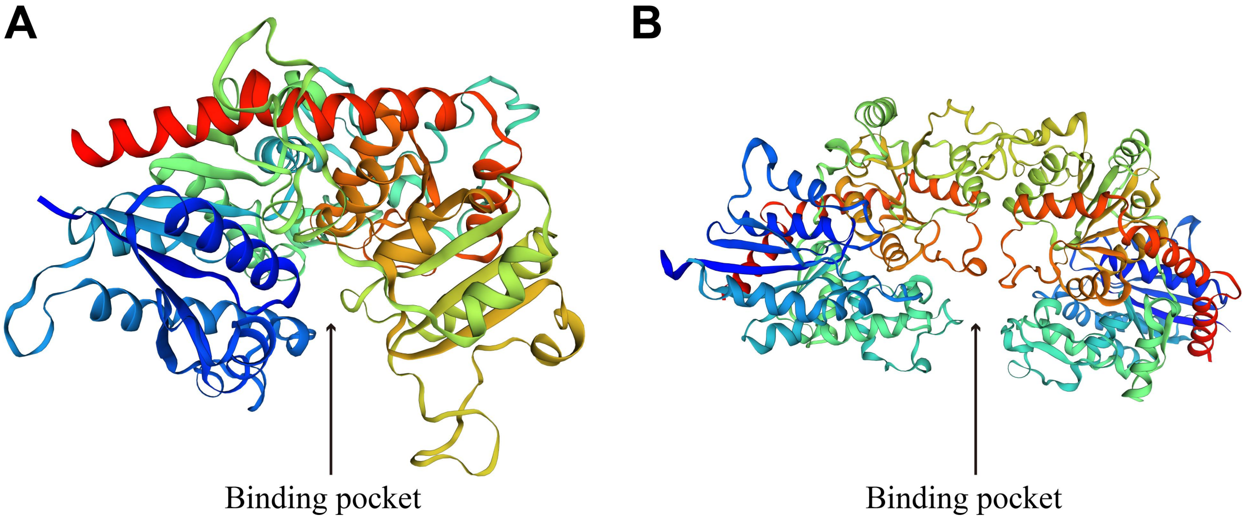

Supplementary Figure 1 Predicted three-dimensional structures of *FmUGT1* and *FmUGT2*. (A): *FmUGT1*; (B): *FmUGT2*. The binding pockets of the *FmUGT1* and *FmUGT2* are identified by black arrows.

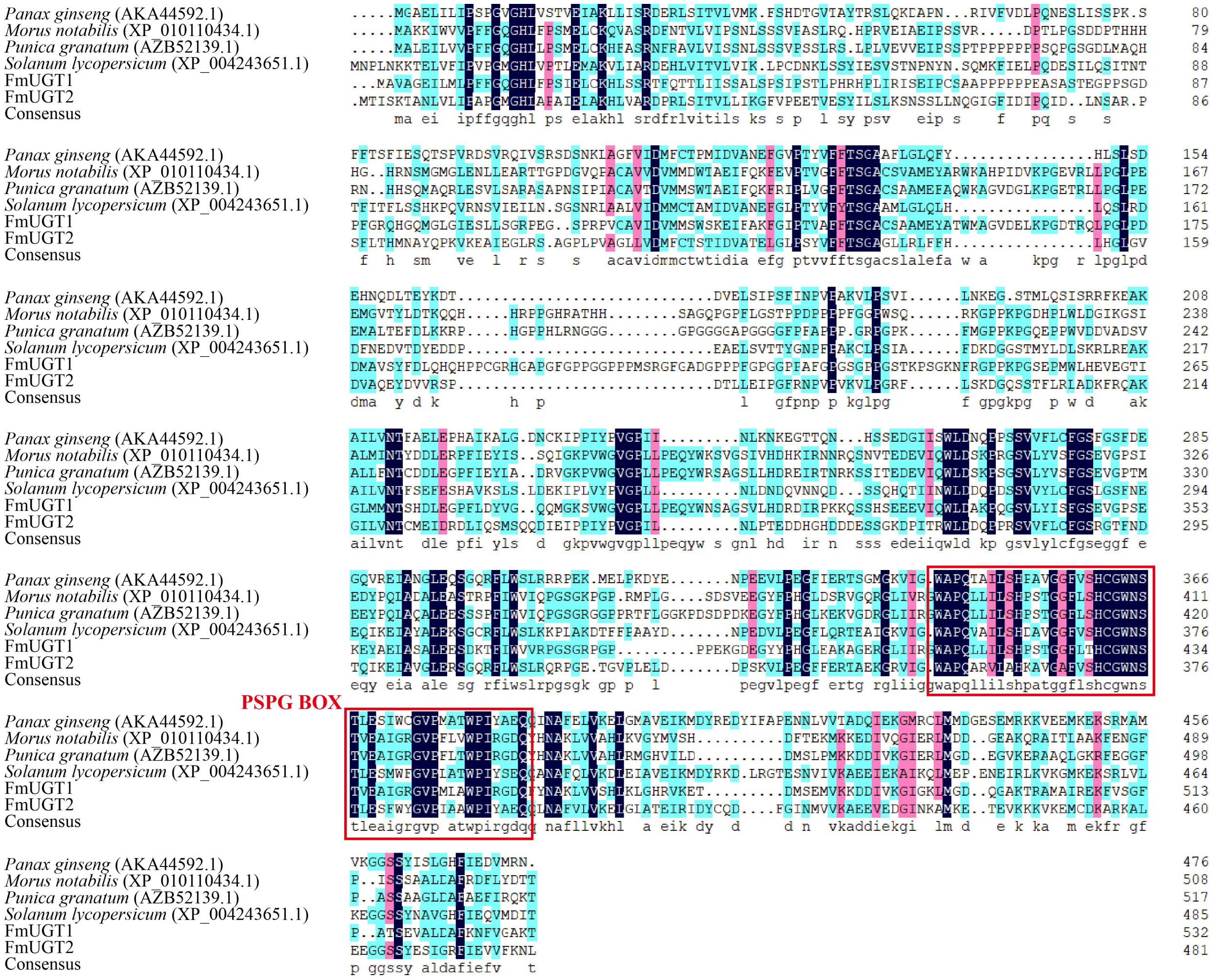

Supplementary Figure 2 Multiple alignment of *FmUGT1* and *FmUGT2* amino acid sequences with other plant UGTs. The UGTs' signature PSPG motifs are enclosed in a red box.

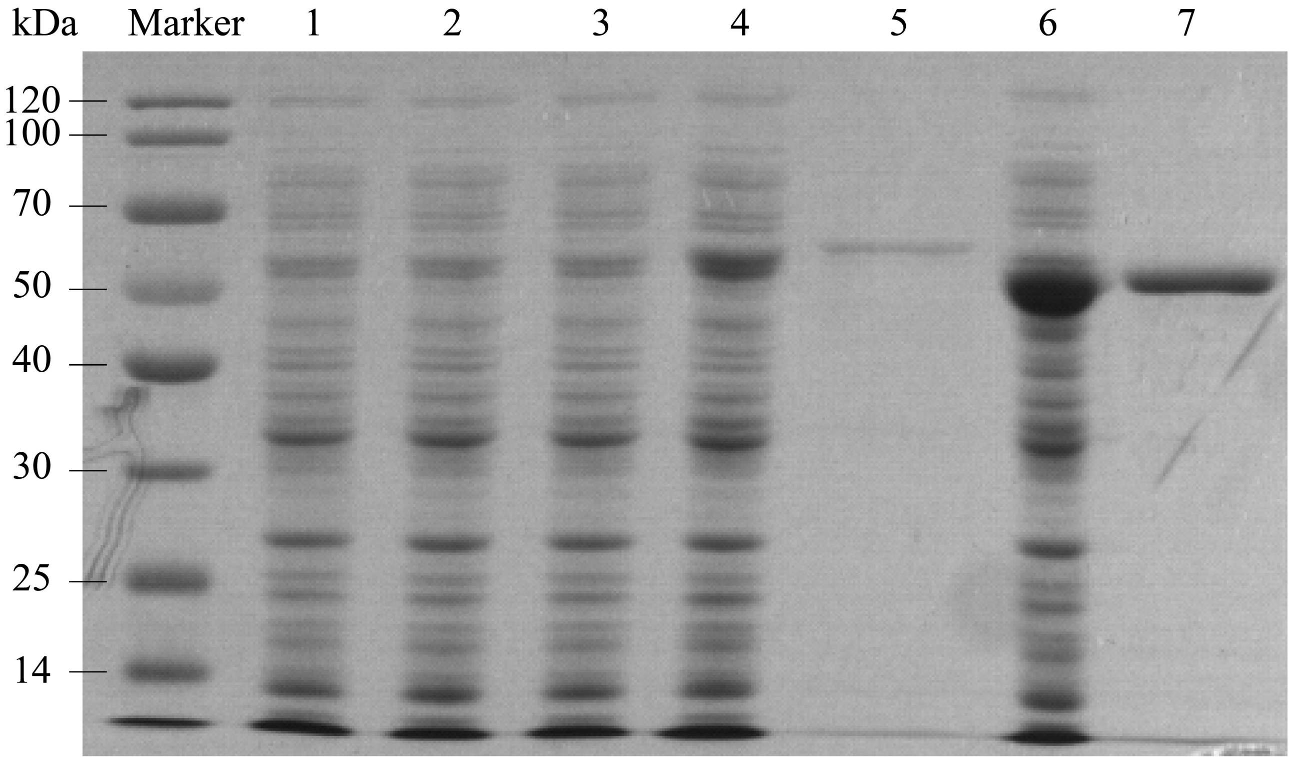

Supplementary Figure 3 10% sodium dodecyl sulphate–polyacrylamide gel electrophoresis (SDS-PAGE) analysis of the expression of recombinant pET-32a-*FmUGT1* and pET-32a-*FmUGT2*. Marker: *Blue Plus*® II Protein Marker (14-120 kDa) (Transgene, China); (1) pET-32a empty vector protein; (2, 3) Uninduced recombinant *FmUGT1* and *FmUGT2* protein; (4, 6) Induced recombinant *FmUGT1* and *FmUGT2* protein; (5, 7) Purified recombinant *FmUGT1* and *FmUGT2* protein.

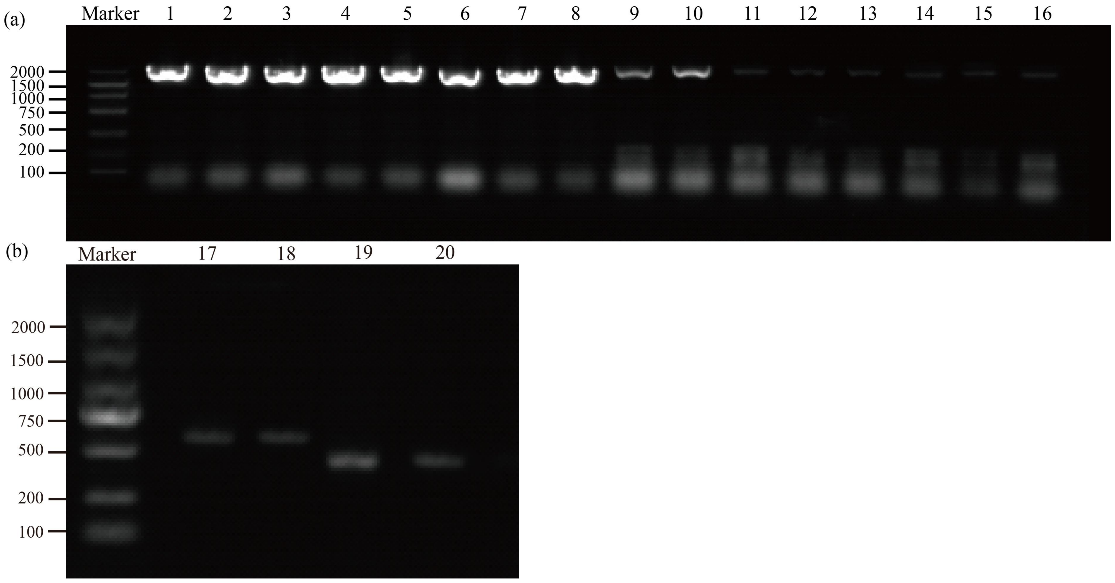

Supplementary Figure 4. PCR analysis of target sequence in recombinant OE/RNAi plant expression vector. (a) For the recombinant vector pK7WG2D-*FmUGT1/2*, 1-8: *FmUGT2*; 9-16: *FmUGT1*; Marker: DNA Marker DL2000 (Zoman Biotechnology, Beijing, China). (b) For the recombinant vector pK7GWIWG2D-*FmUGT1i/2i*, 17: *FmUGT1i*; 18: *FmUGT2i*; 19: antisense-*FmUGT11i*; 20: antisense-*FmUGT36i*.

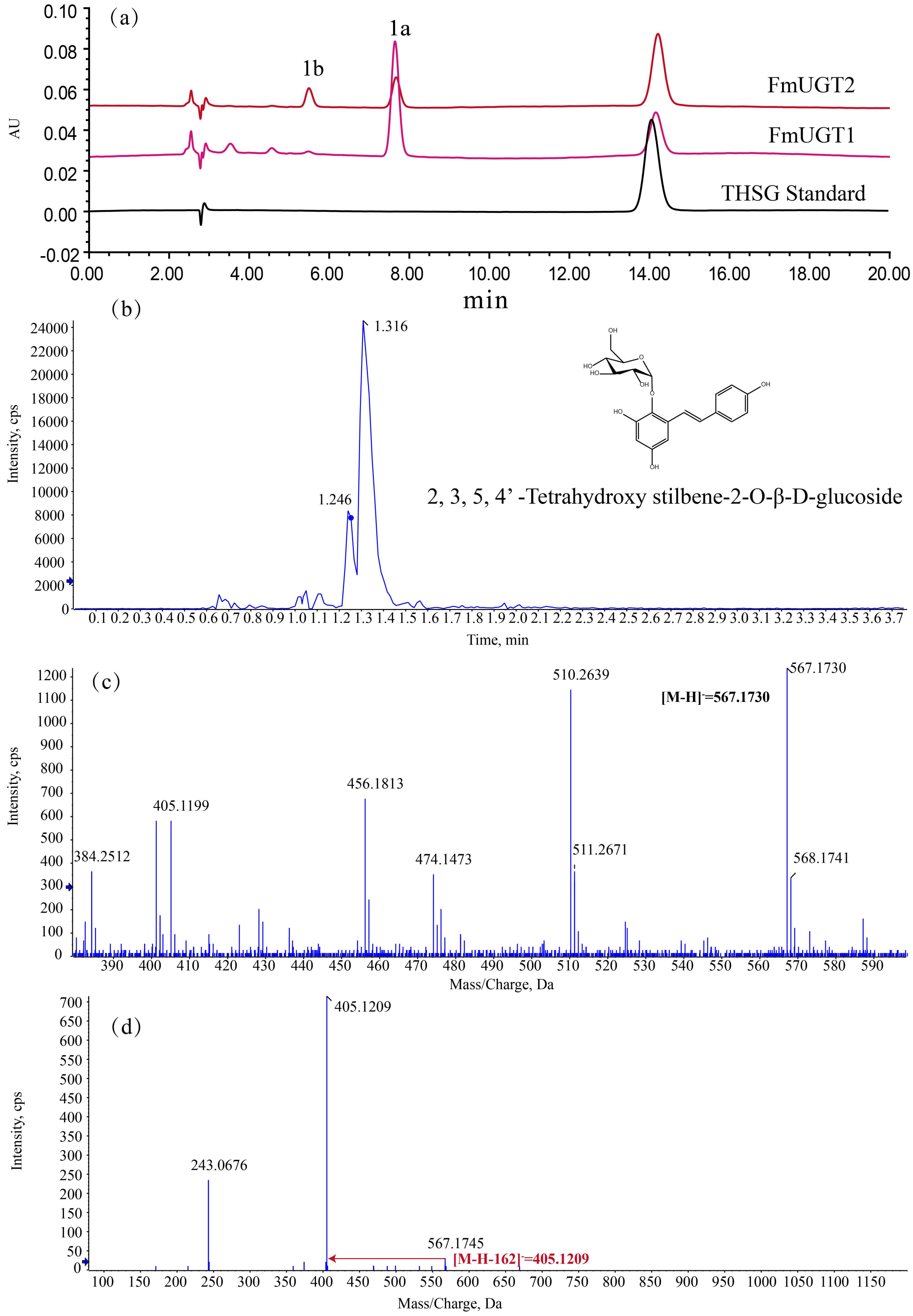

Supplementary Figure 5. LC-MS/MS analysis of FmUGTs catalytic reaction mixture for substrate 1. (a) HPLC chromatogram of 1 and enzymatic products; (b) The selected ion current detection of the glycosylation products quasi-molecular ion peak at m/z 567.1730 $[M-H]^-$; (c) and (d) (-)-ESI-MS and MS/MS spectra for 1b.

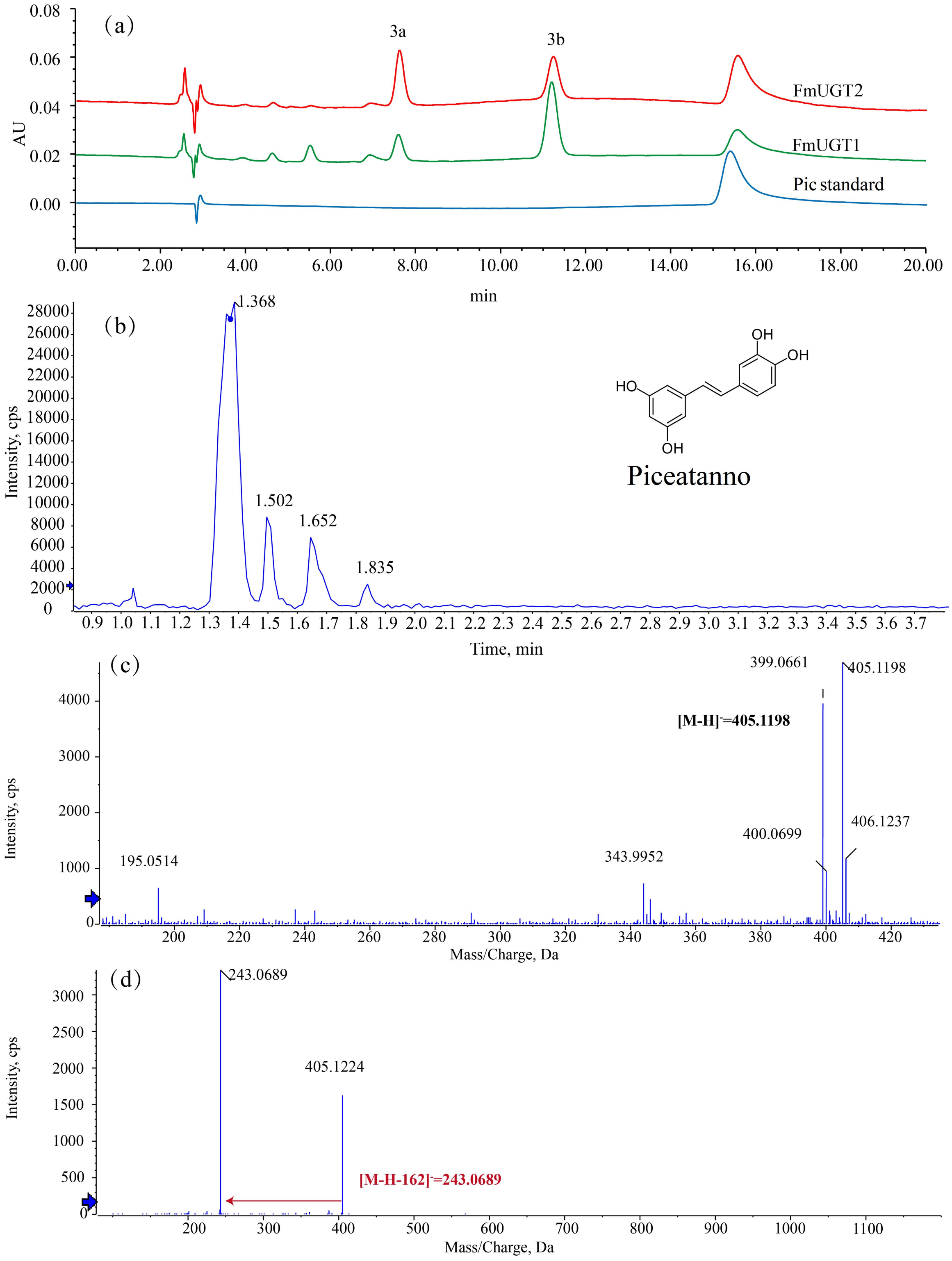

Supplementary Figure 6. LC-MS/MS analysis of FmUGTs catalytic reaction mixture for substrate 3. (a) HPLC chromatogram of 3 and enzymatic products; (b) The selected ion current detection of the glycosylation products quasi-molecular ion peak at m/z 405.1198 $[M-H]^-$; (c) and (d) (-)-ESI-MS and MS/MS spectra for 3a.

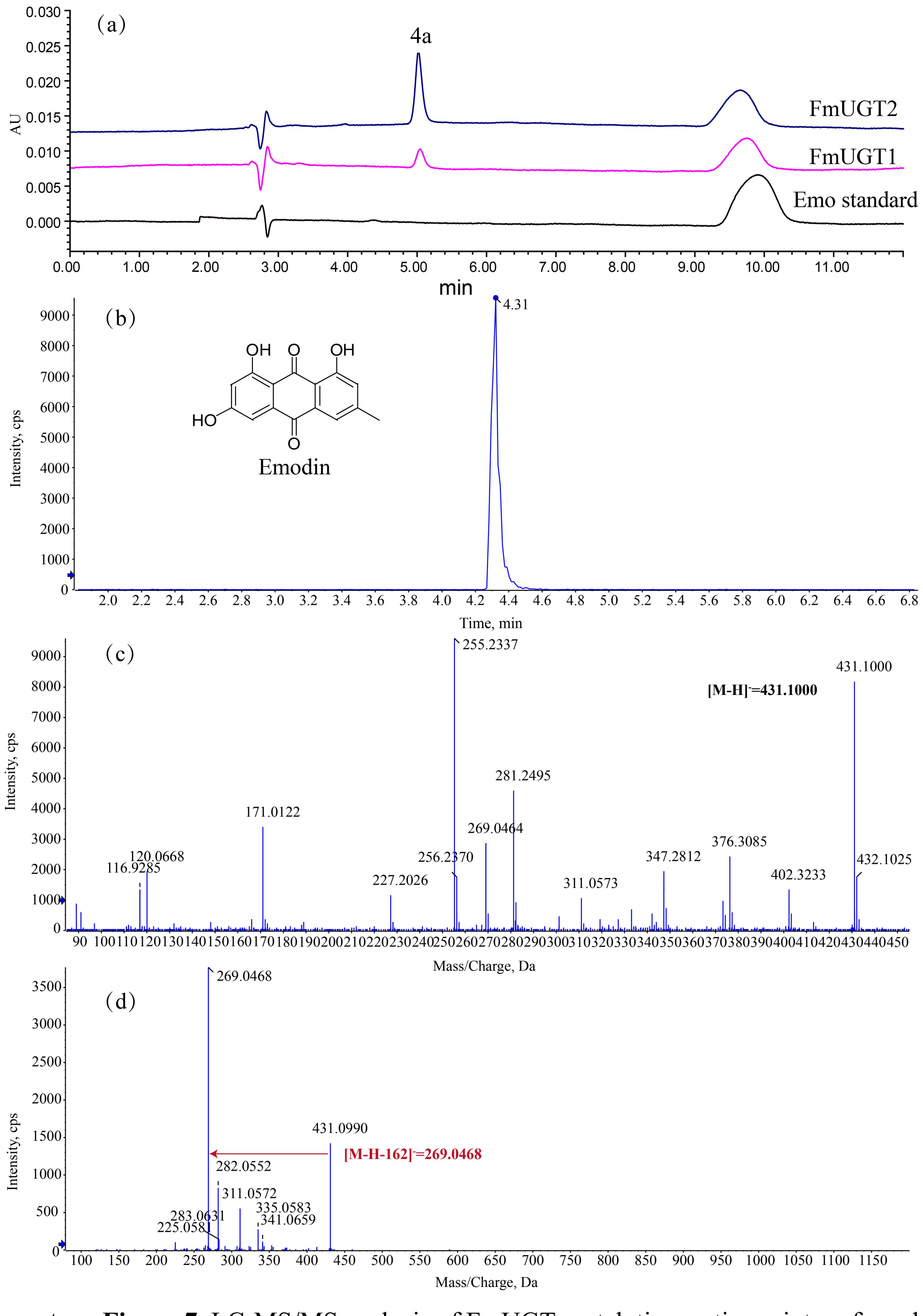

Supplementary Figure 7. LC-MS/MS analysis of FmUGTs catalytic reaction mixture for substrate 4. (a) HPLC chromatogram of 4 and enzymatic product; (b) The selected ion current detection of the glycosylation products quasi-molecular ion peak at m/z 431.1000 $[M-H]^-$; (c) and (d) (-)-ESI-MS and MS/MS spectra for 4a.

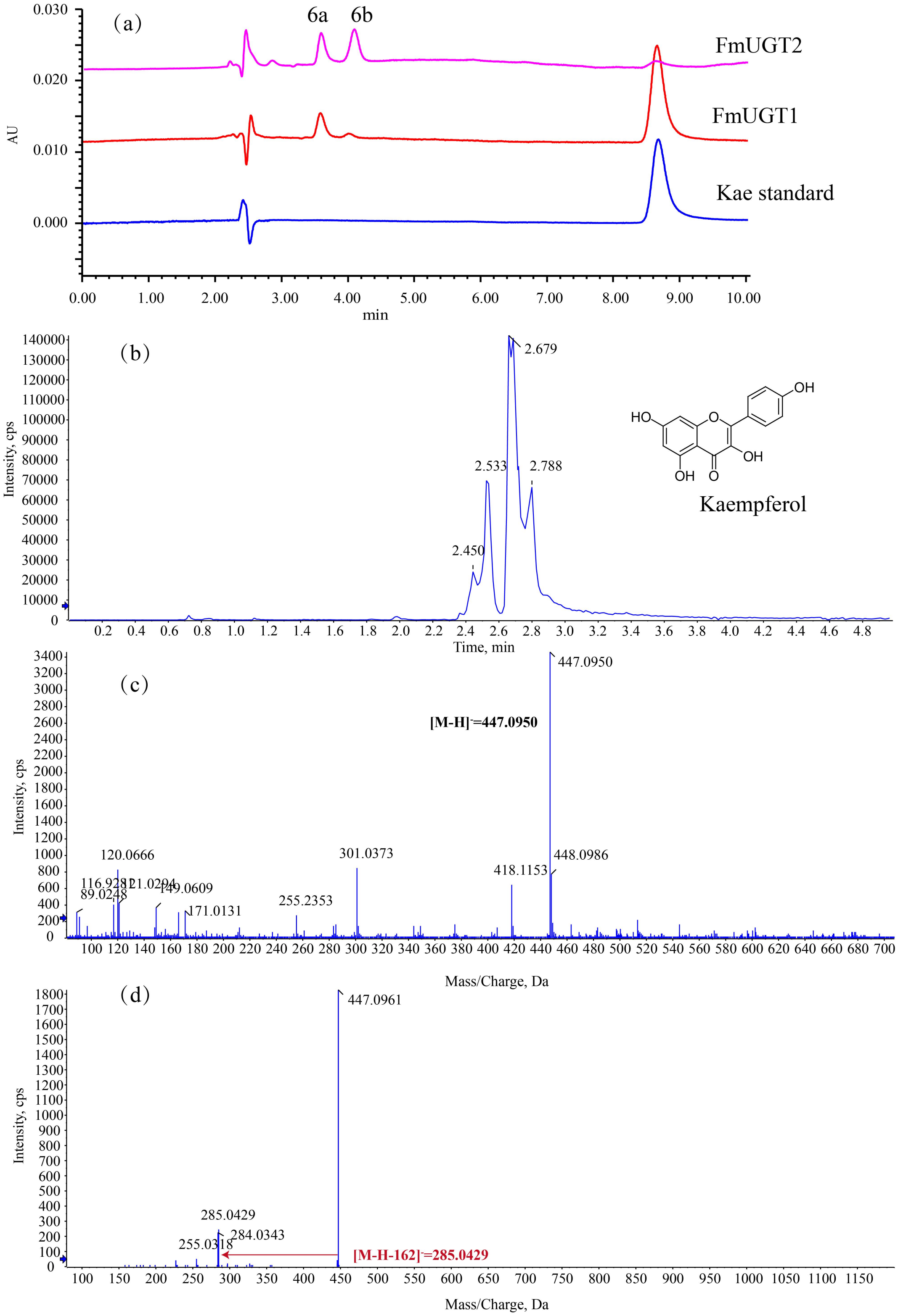

Supplementary Figure 8. LC-MS/MS analysis of FmUGTs catalytic reaction mixture for substrate 6. (a) HPLC chromatogram of 6 and enzymatic products; (b) The selected ion current detection of the glycosylation products quasi-molecular ion peak at m/z 447.0950 $[M-H]^-$; (c) and (d) (-)-ESI-MS and MS/MS spectra for 6a.

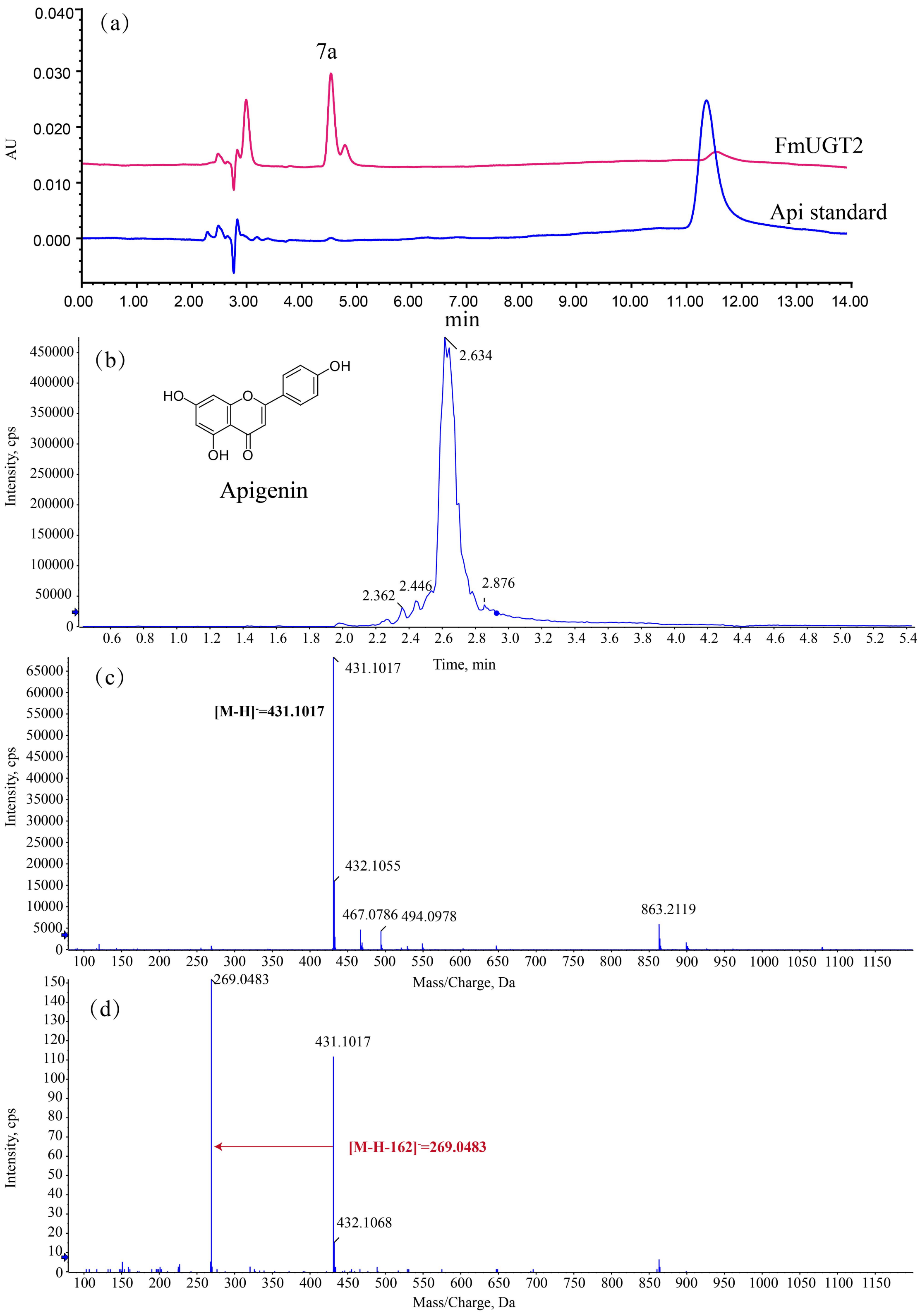

Supplementary Figure 9. LC-MS/MS analysis of FmUGTs catalytic reaction mixture for substrate 7. (a) HPLC chromatogram of 7 and enzymatic product; (b) The selected ion current detection of the glycosylation products quasi-molecular ion peak at m/z 431.1017 $[M-H]^-$; (c) and (d) (-)-ESI-MS and MS/MS spectra for 7a.

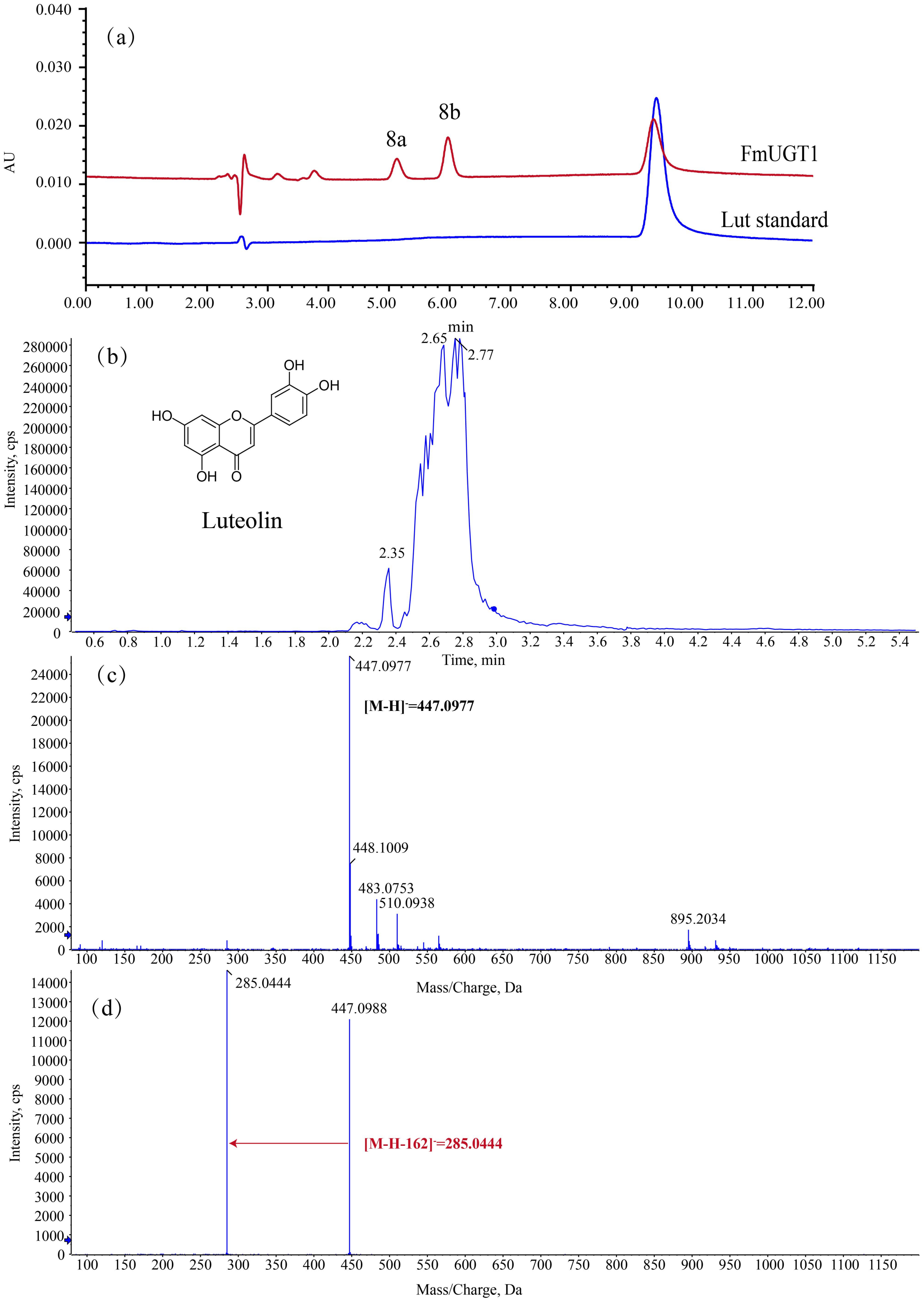

Supplementary Figure 10. LC-MS/MS analysis of FmUGTs catalytic reaction mixture for substrate 8. (a) HPLC chromatogram of 8 and enzymatic product; (b) The selected ion current detection of the glycosylation products quasi-molecular ion peak at m/z 447.0977 $[M-H]^-$; (c) and (d) (-)-ESI-MS and MS/MS spectra for 8a.

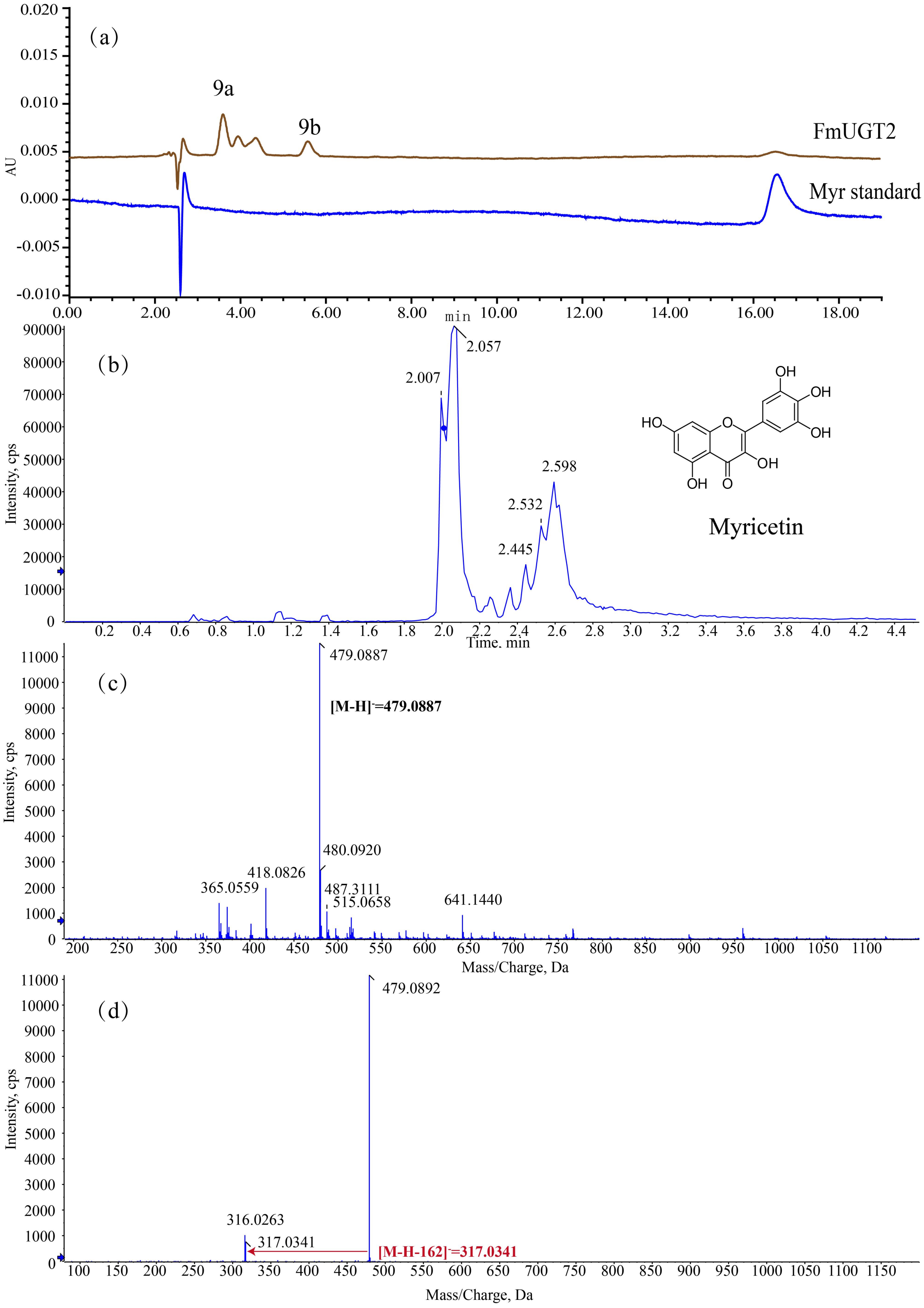

Supplementary Figure 11. LC-MS/MS analysis of FmUGTs catalytic reaction mixture for substrate 9. (a) HPLC chromatogram of 9 and enzymatic product; (b) The selected ion current detection of the glycosylation products quasi-molecular ion peak at m/z 479.0887 $[M-H]^-$; (c) and (d) (-)-ESI-MS and MS/MS spectra for 9a.

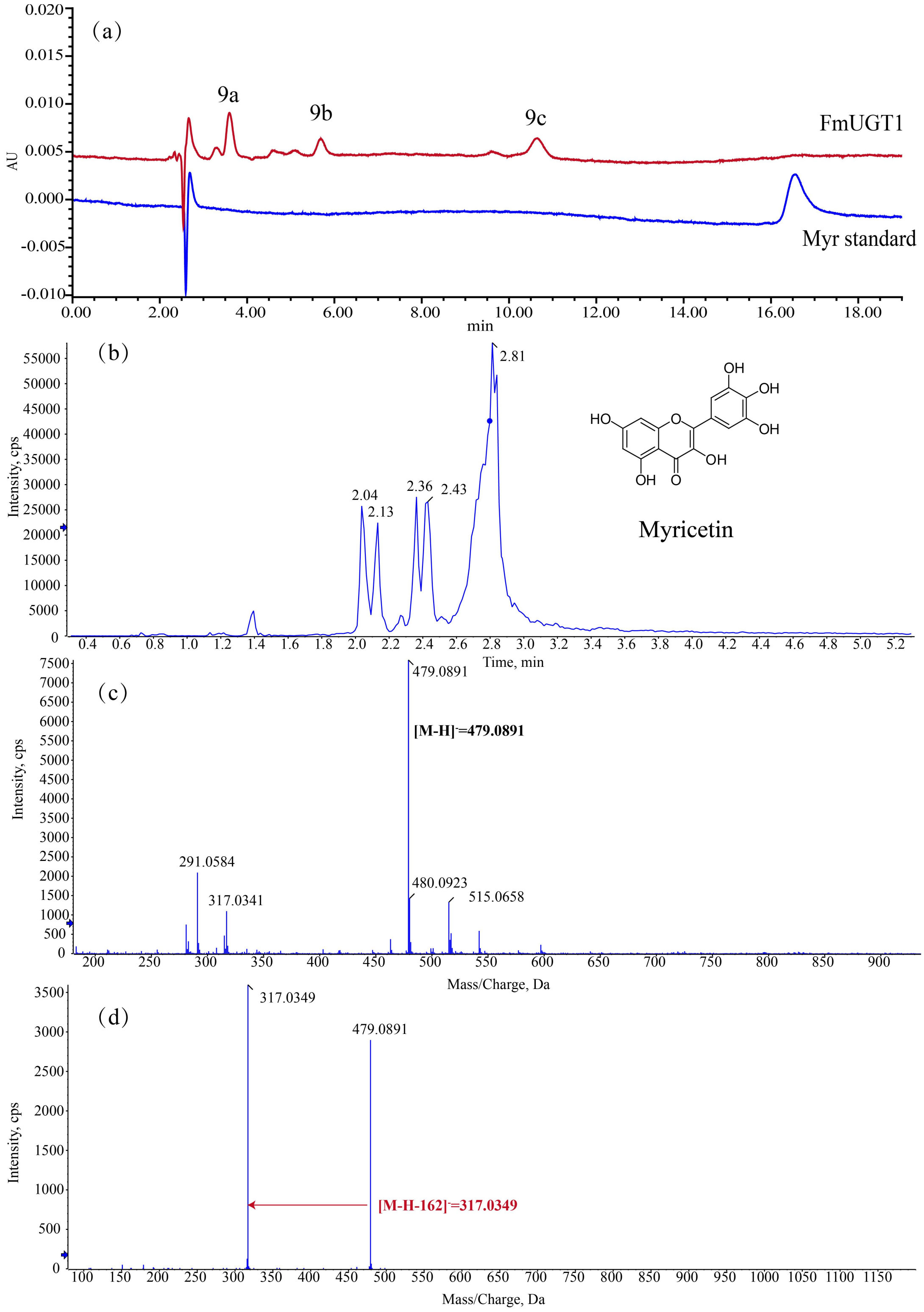

Supplementary Figure 12. LC-MS/MS analysis of FmUGTs catalytic reaction mixture for substrate 9. (a) HPLC chromatogram of 9 and enzymatic product; (b) The selected ion current detection of the glycosylation products quasi-molecular ion peak at m/z 479.0891 $[M-H]^-$; (c) and (d) (-)-ESI-MS and MS/MS spectra for 9c.

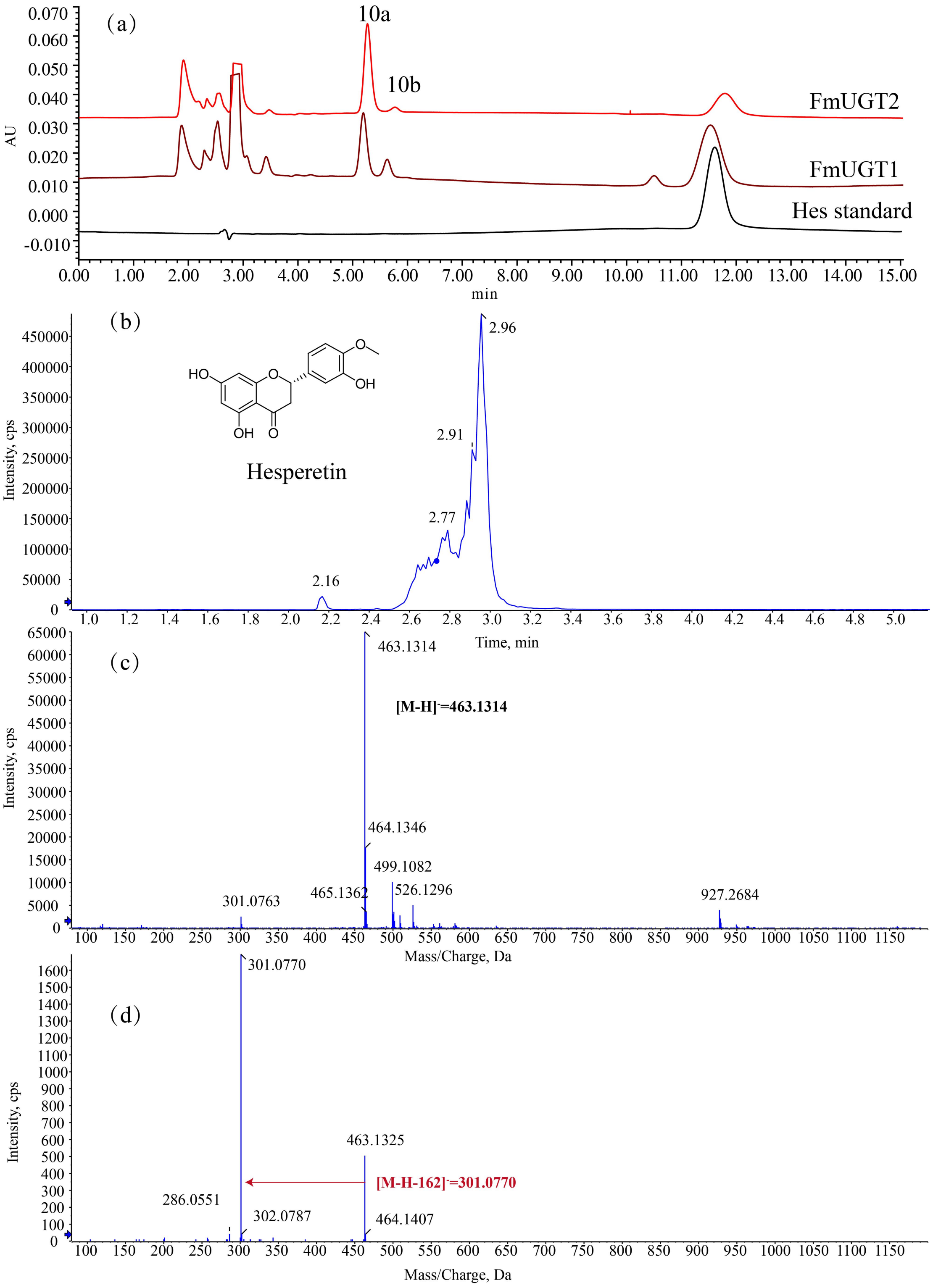

Supplementary Figure 13. LC-MS/MS analysis of FmUGTs catalytic reaction mixture for substrate 10. (a) HPLC chromatogram of 10 and enzymatic product; (b) The selected ion current detection of the glycosylation products quasi-molecular ion peak at m/z 463.1314 $[M-H]^-$; (c) and (d) (-)-ESI-MS and MS/MS spectra for 10a.

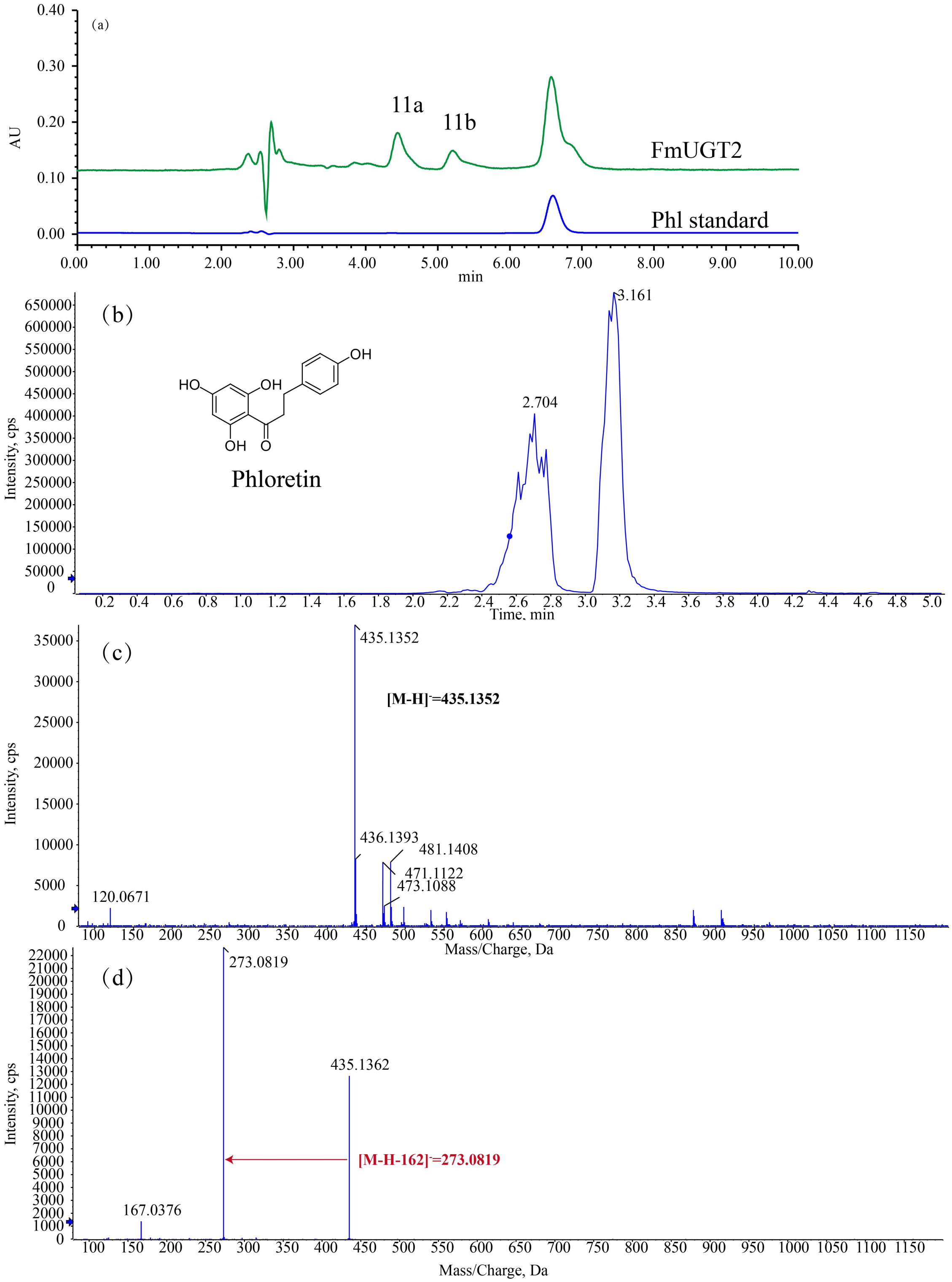

Supplementary Figure 14. LC-MS/MS analysis of FmUGTs catalytic reaction mixture for substrate 11. (a) HPLC chromatogram of 11 and enzymatic product; (b) The selected ion current detection of the glycosylation products quasi-molecular ion peak at m/z 435.1352 $[M-H]^-$; (c) and (d) (-)-ESI-MS and MS/MS spectra for 11a.

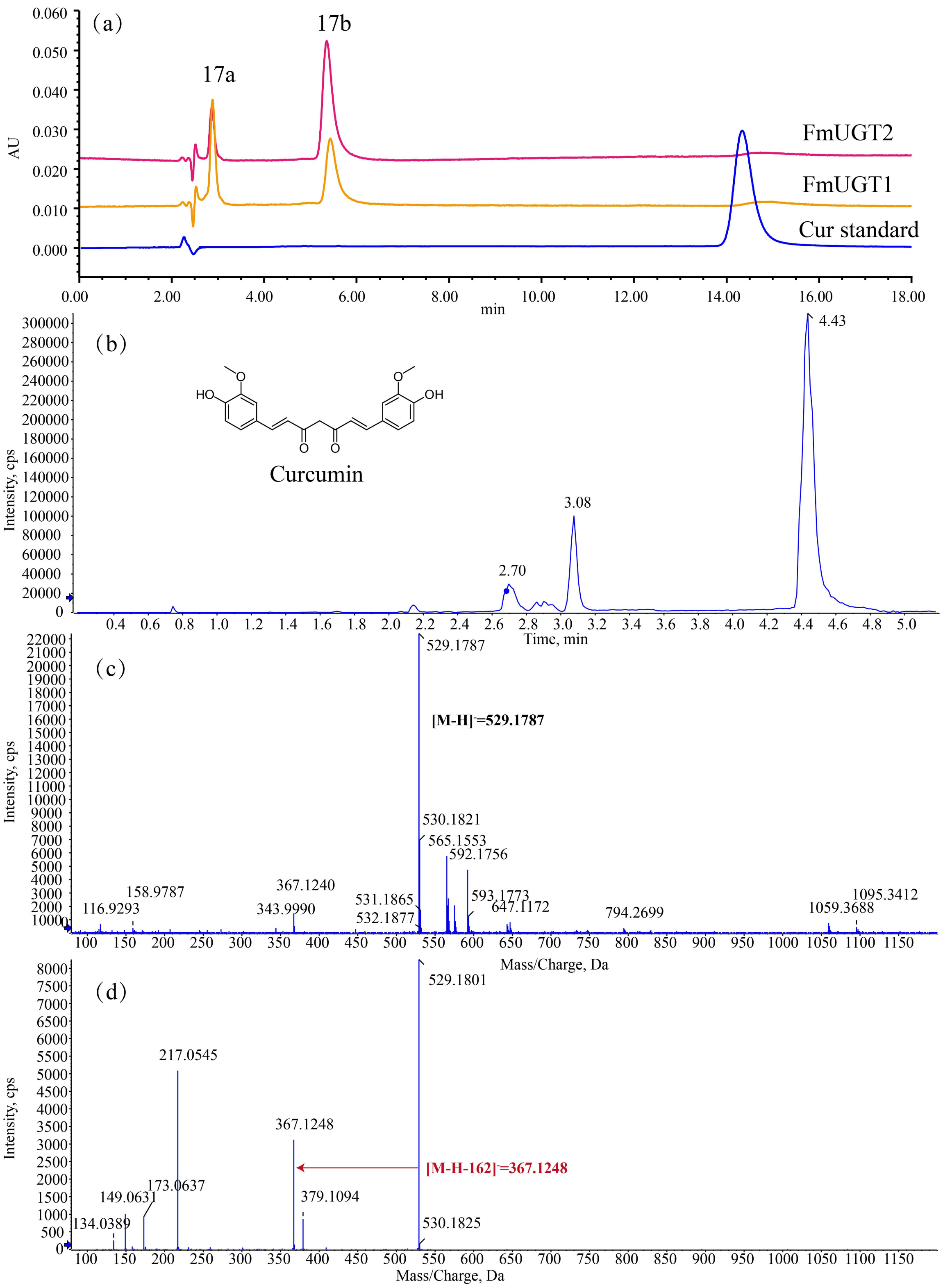

Supplementary Figure 15. LC-MS/MS analysis of FmUGTs catalytic reaction mixture for substrate 12. (a) HPLC chromatogram of 12 and enzymatic product; (b) The selected ion current detection of the glycosylation products quasi-molecular ion peak at m/z 529.1787 $[M-H]^-$; (c) and (d) (-)-ESI-MS and MS/MS spectra for 12b.

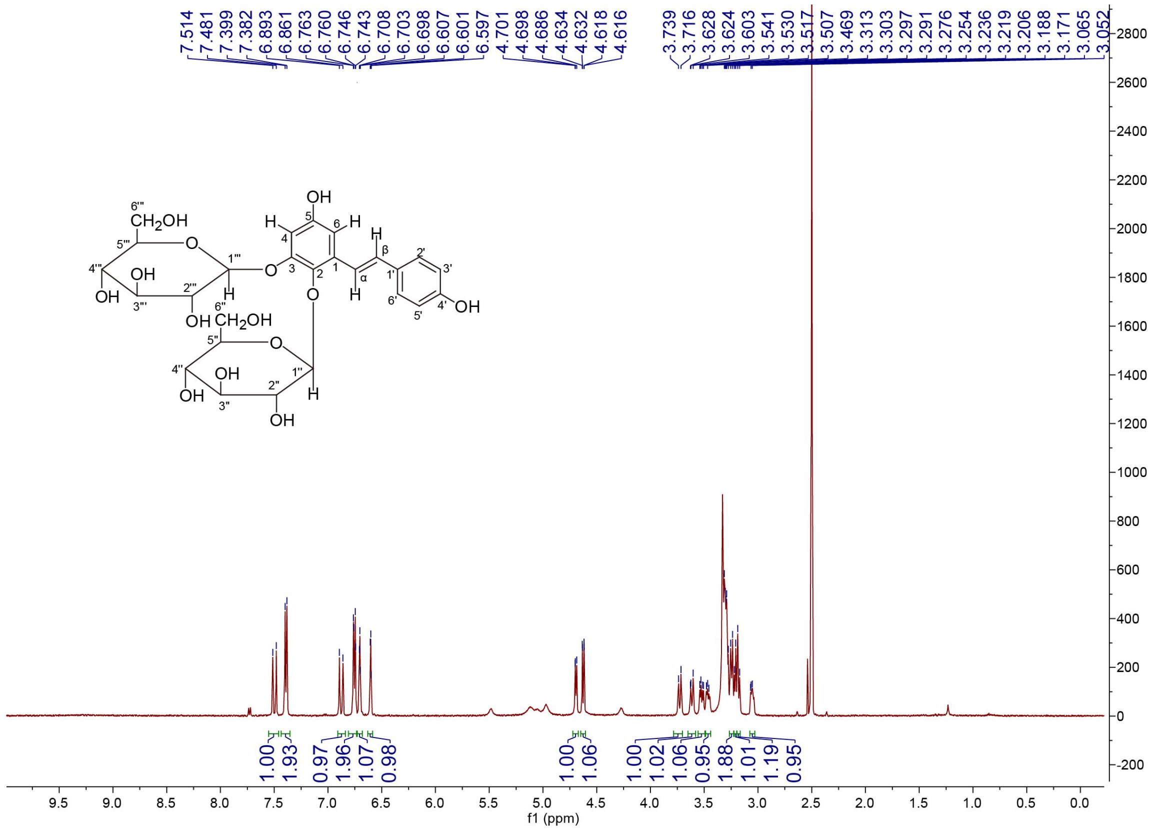

Supplementary Figure 16. ^{1}H NMR spectrum of compound 1a (DMSO-d_6, 500 MHz).

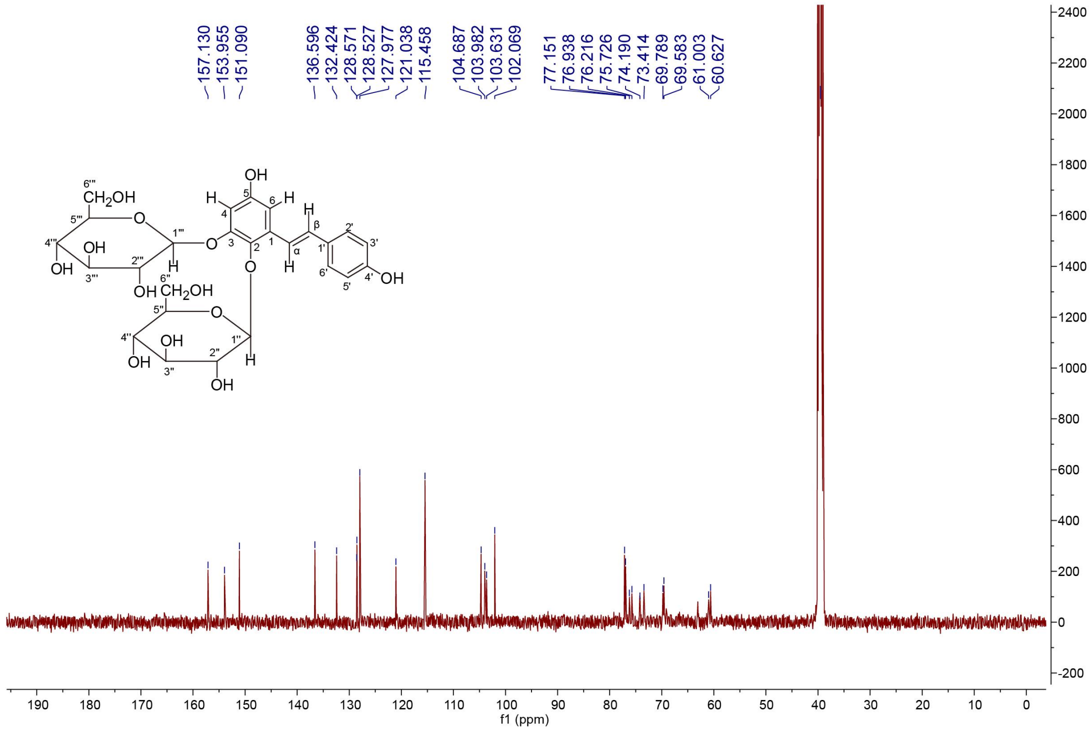

Supplementary Figure 17. ^{13}C NMR spectrum of compound 1a (DMSO-d_6, 126 MHz).

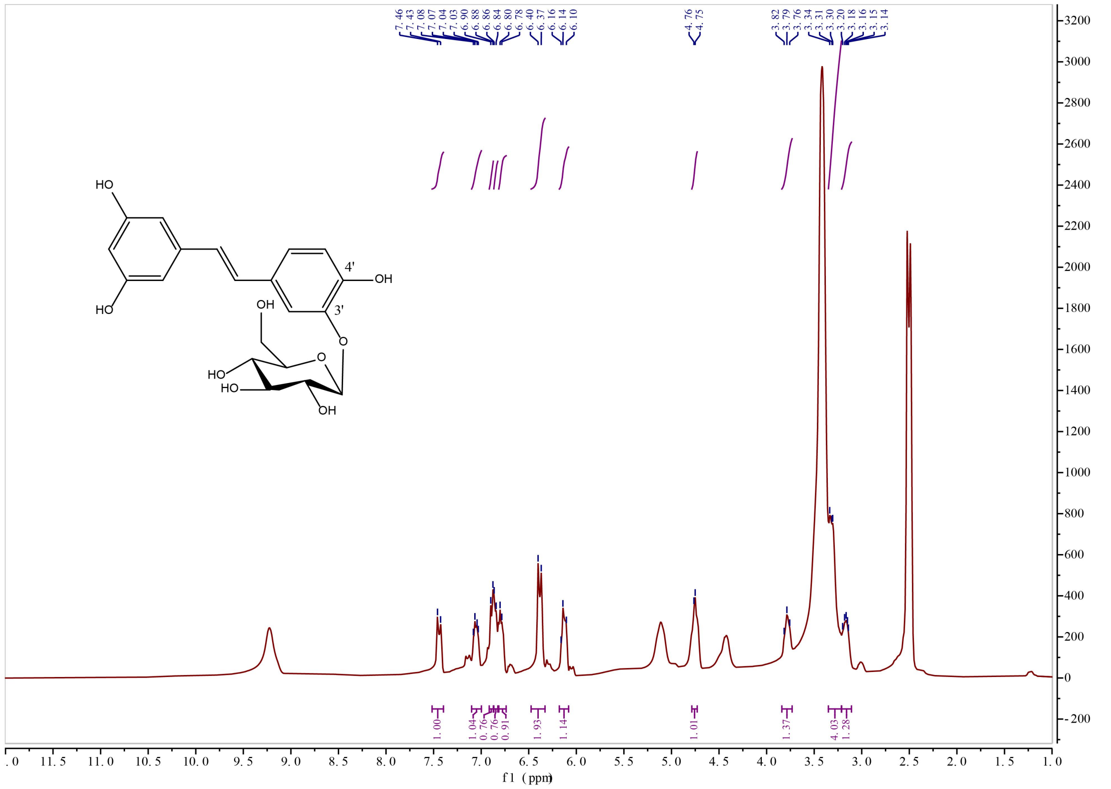

Supplementary Figure 18. ^{1}H NMR spectrum of compound 3a (DMSO-d_6, 500 MHz).

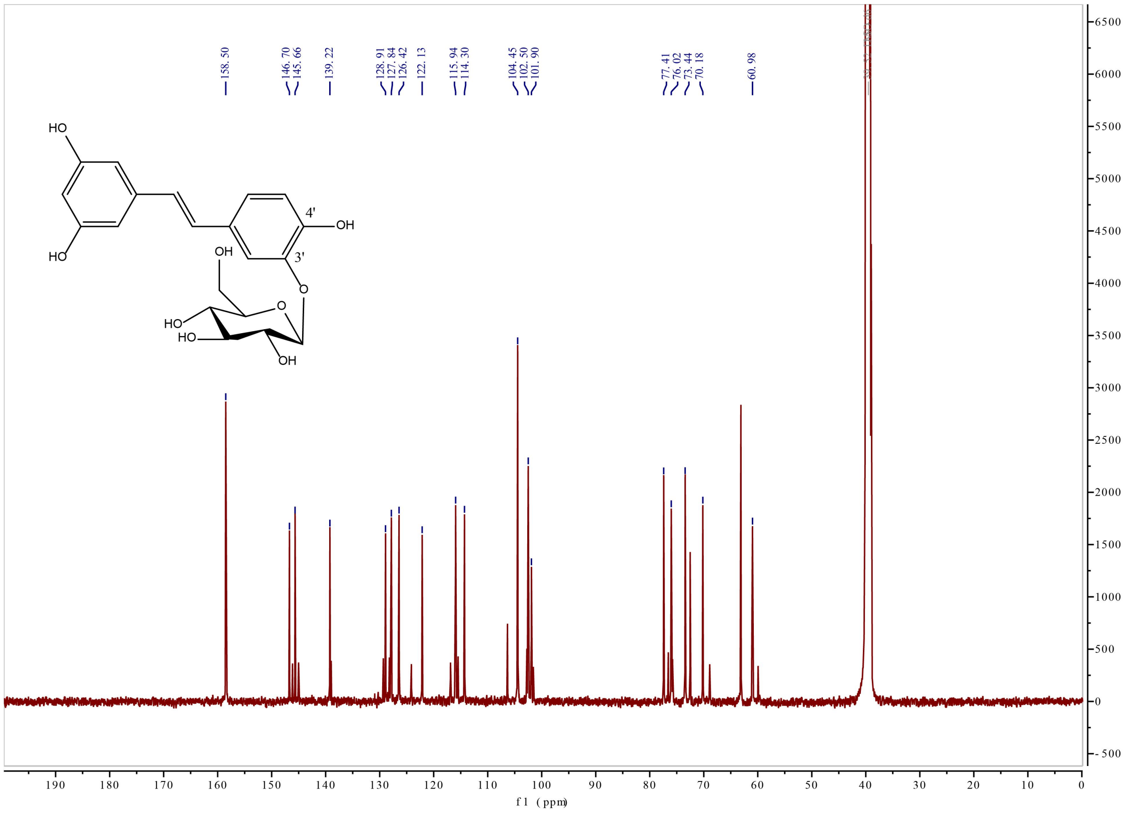

Supplementary Figure 19. ^{13}C NMR spectrum of compound 3a (DMSO-d_6, 126 MHz).

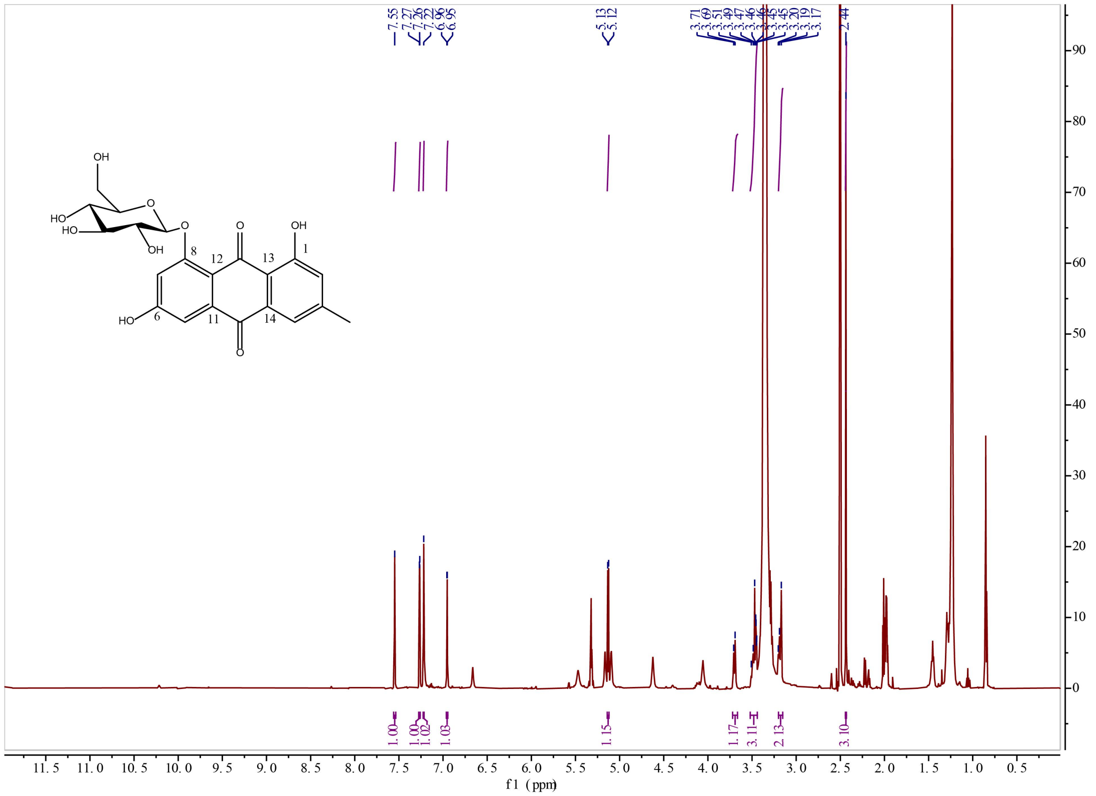

Supplementary Figure 20. ^{1}H NMR spectrum of compound 4a (DMSO-d_6,700 MHz).

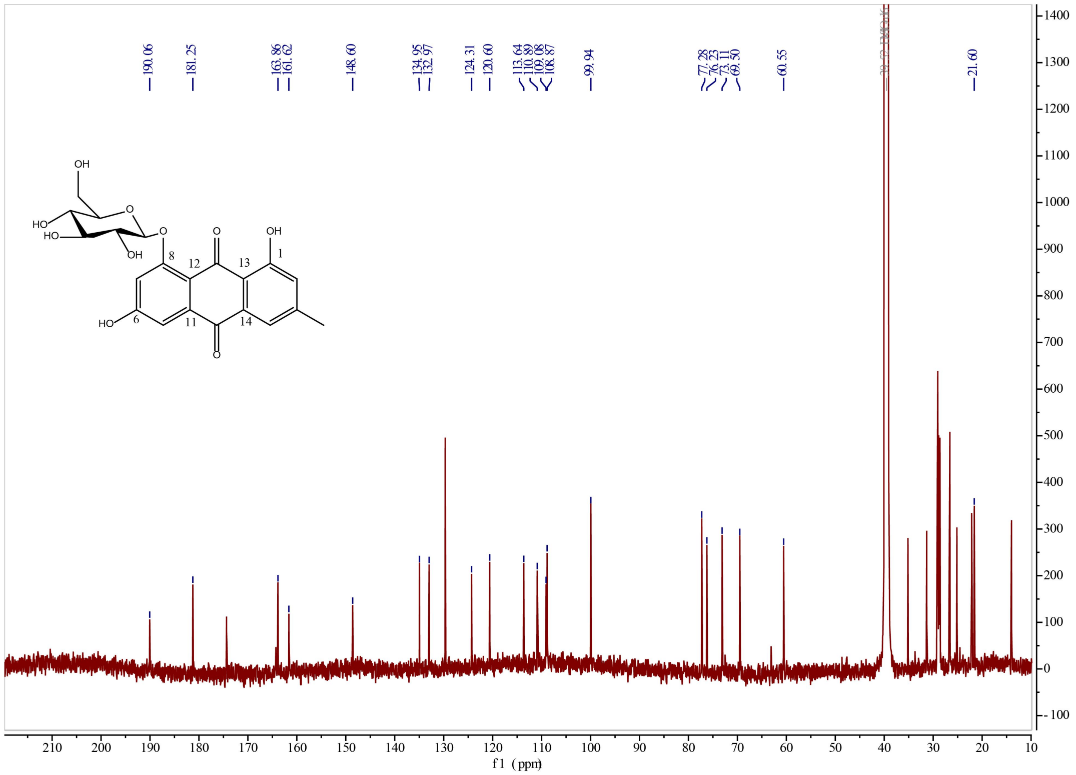

Supplementary Figure 21. ^{13}C NMR spectrum of compound 4a (DMSO-d_6, 176 MHz).

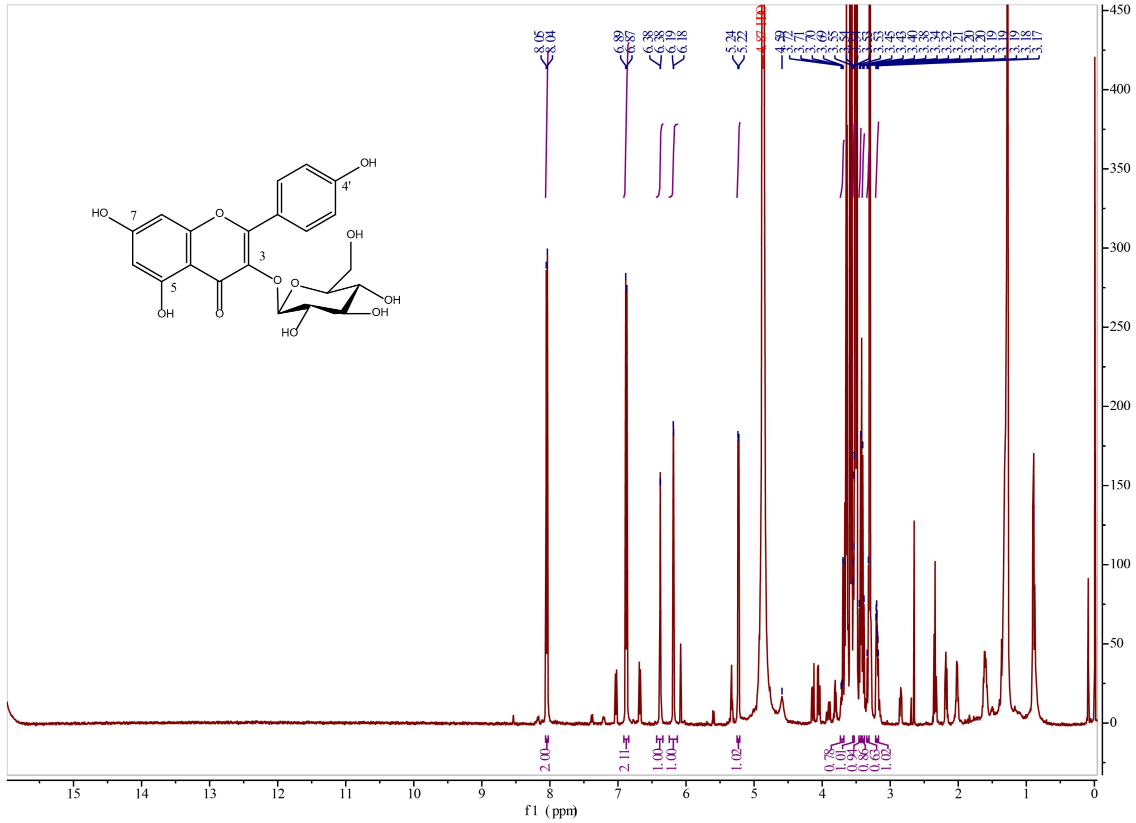

Supplementary Figure 22. ^{1}H NMR spectrum of compound 6a (Methanol-d_4, 500 MHz).

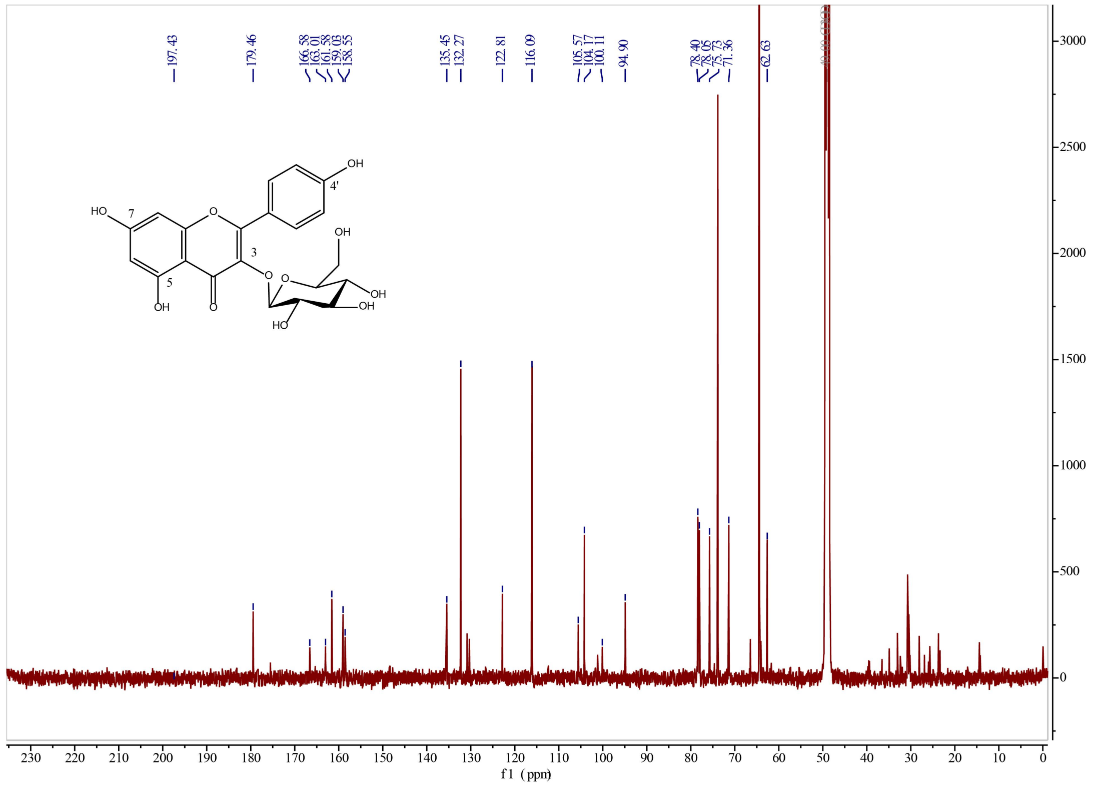

Supplementary Figure 23. ^{13}C NMR spectrum of compound 6a (Methanol-d_4, 126 MHz).

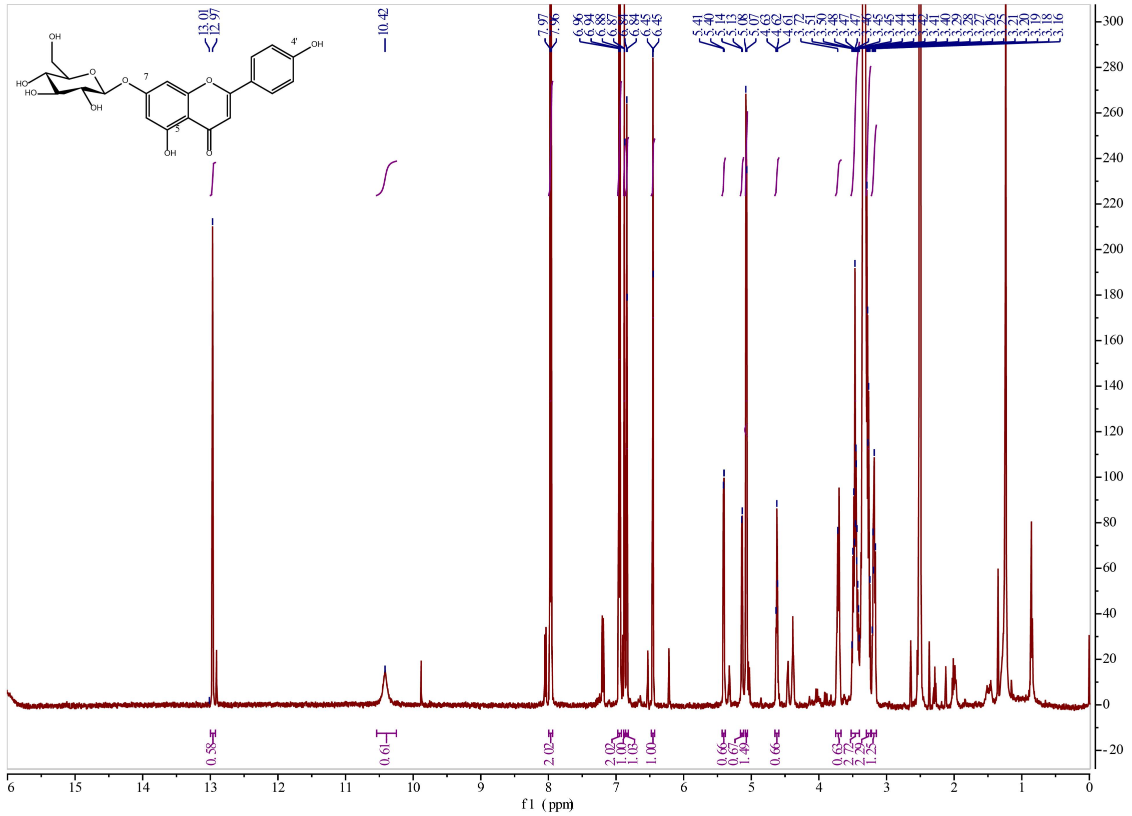

Supplementary Figure 24. ^{1}H NMR spectrum of compound 7a (DMSO-d_6, 500 MHz).

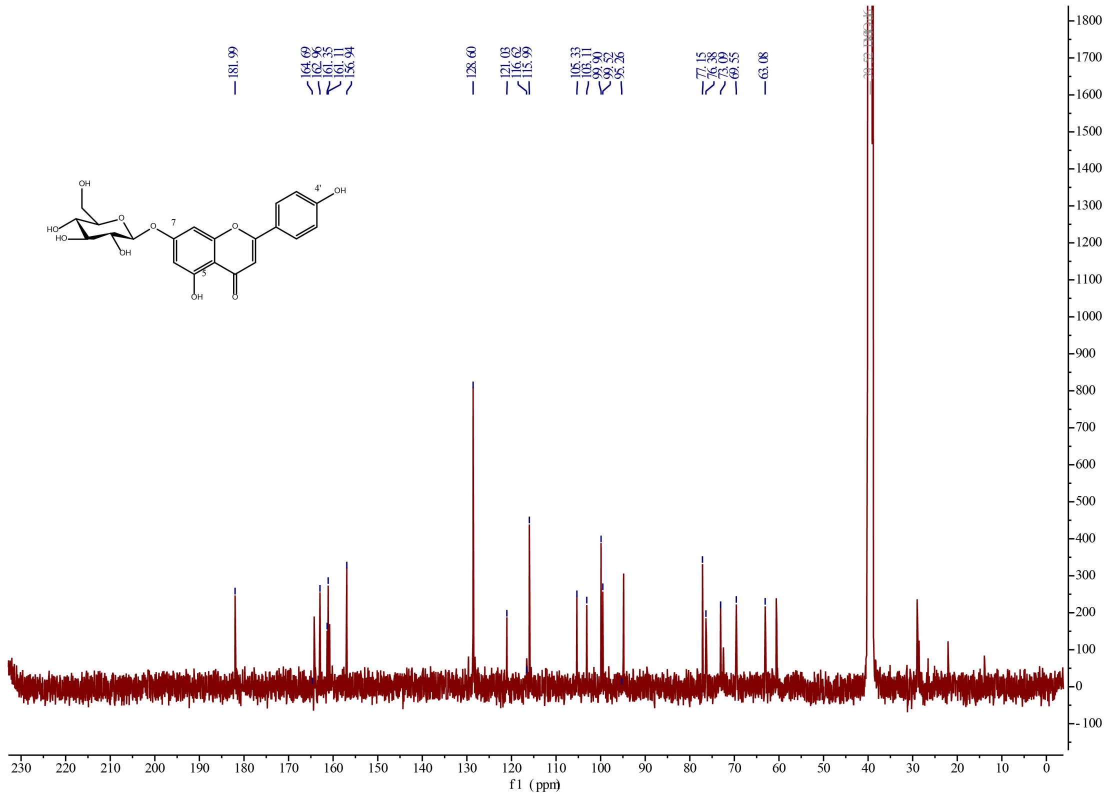

Supplementary Figure 25. ^{13}C NMR spectrum of compound 7a (DMSO-d_6, 126 MHz).

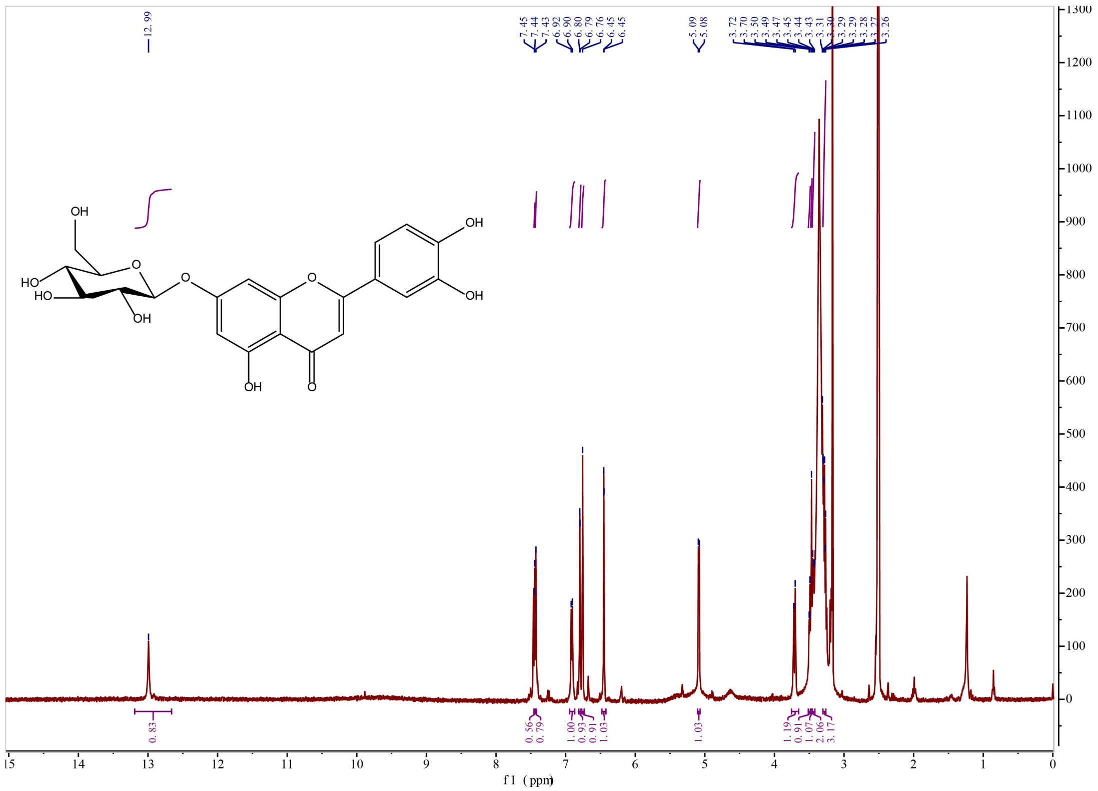

Supplementary Figure 26. ^{1}H NMR spectrum of compound 8b (DMSO-d_6, 500 MHz).

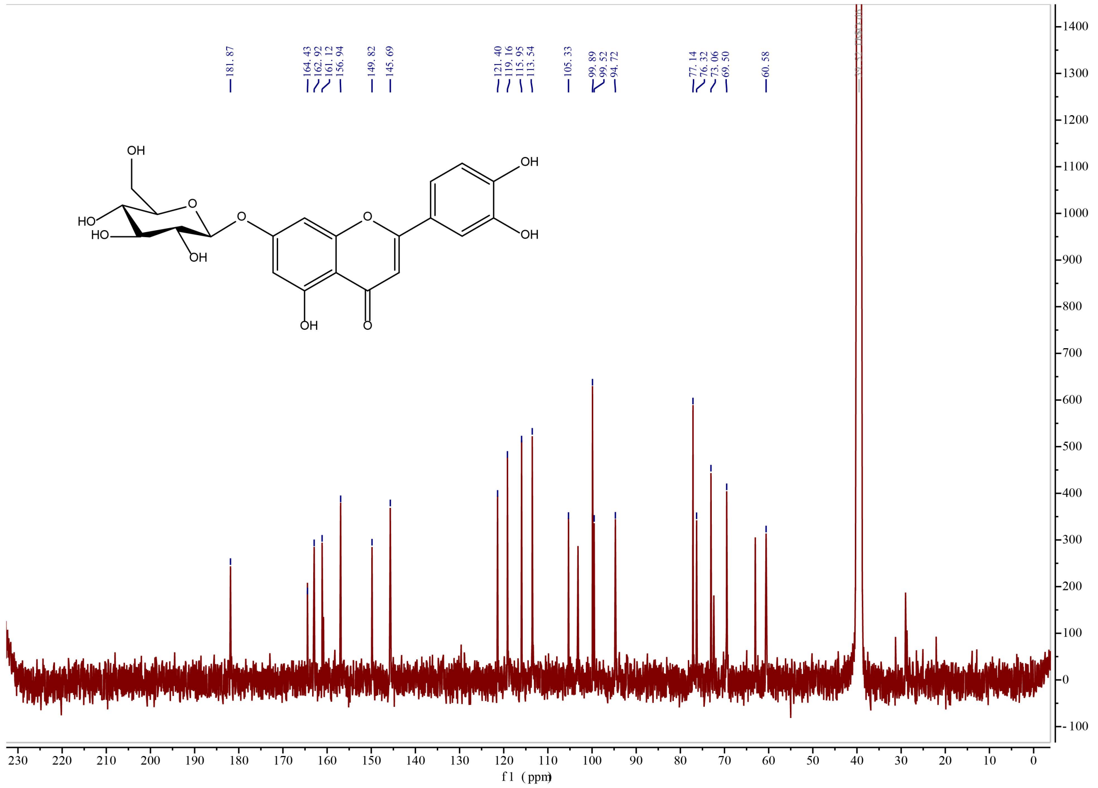

Supplementary Figure 27. ^{13}C NMR spectrum of compound 8b (DMSO-d_6, 126 MHz).

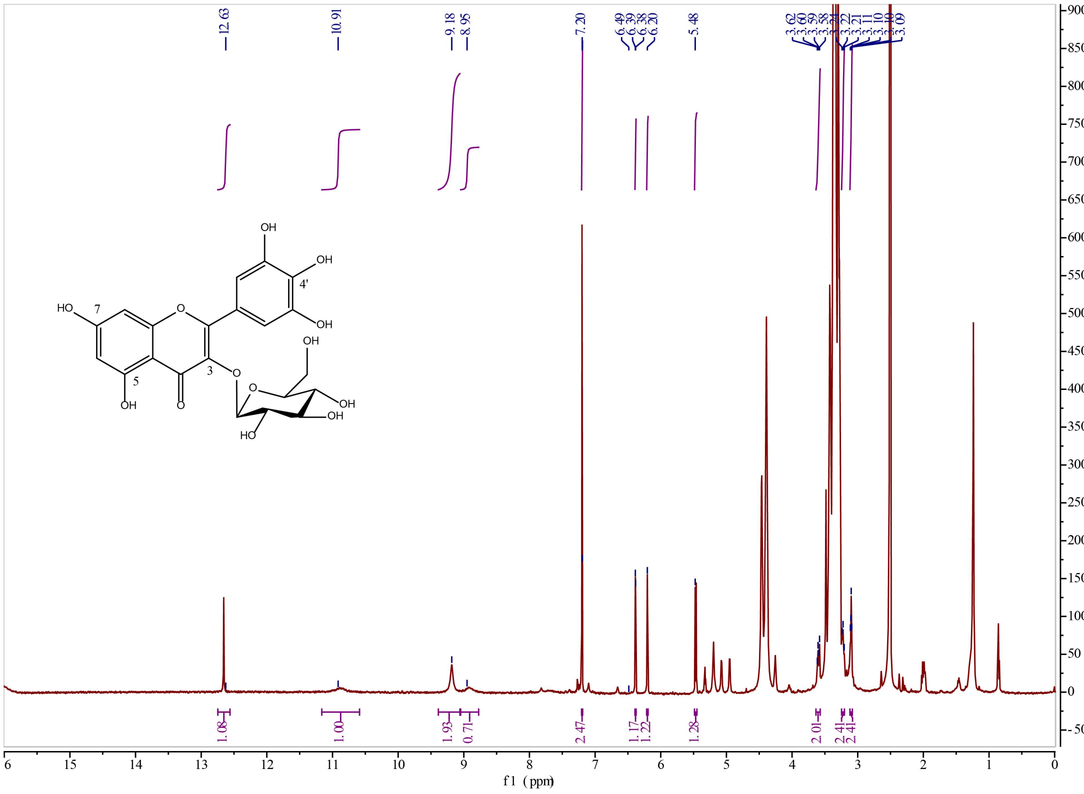

Supplementary Figure 28. ^{1}H NMR spectrum of compound 9a (DMSO-d_6, 500 MHz).

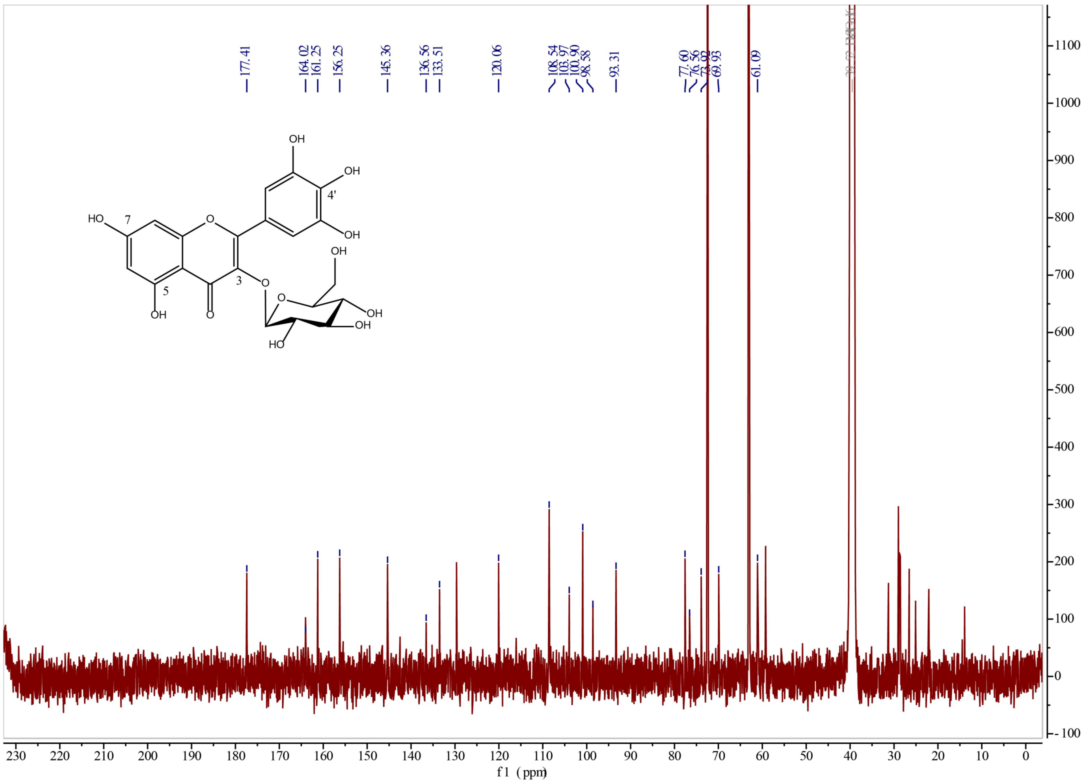

Supplementary Figure 29. ^{13}C NMR spectrum of compound 9a (DMSO-d_6, 126 MHz).

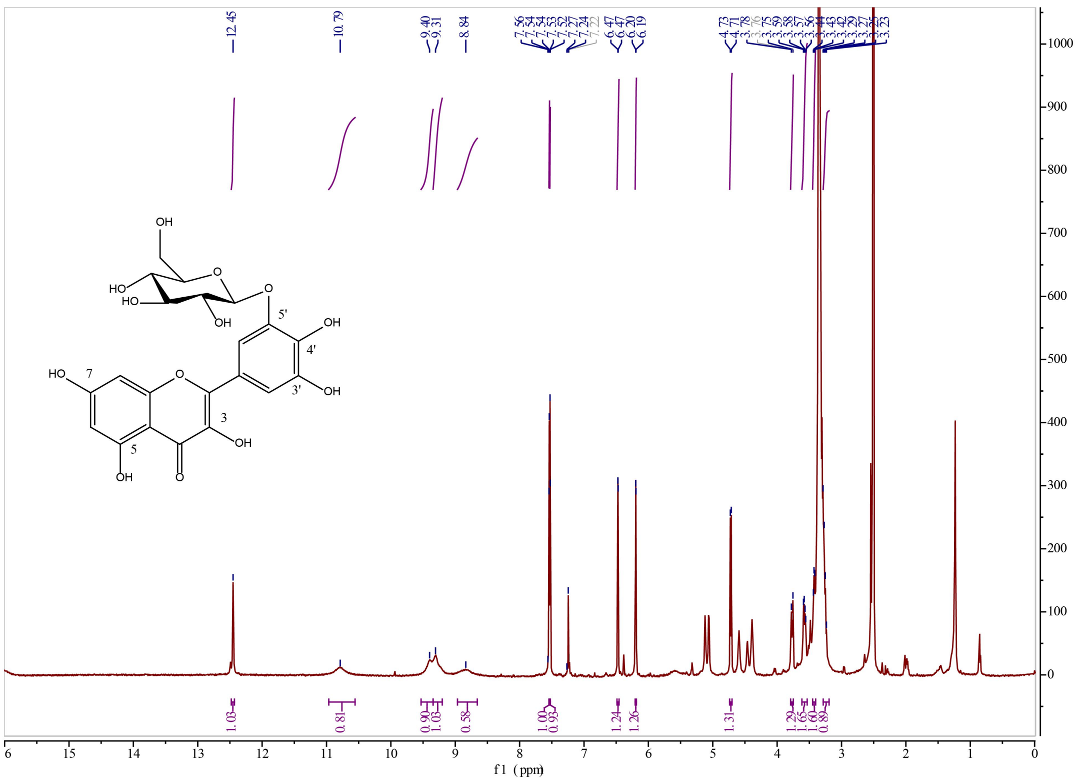

Supplementary Figure 30. ^{1}H NMR spectrum of compound 9c (DMSO-d_6, 500 MHz).

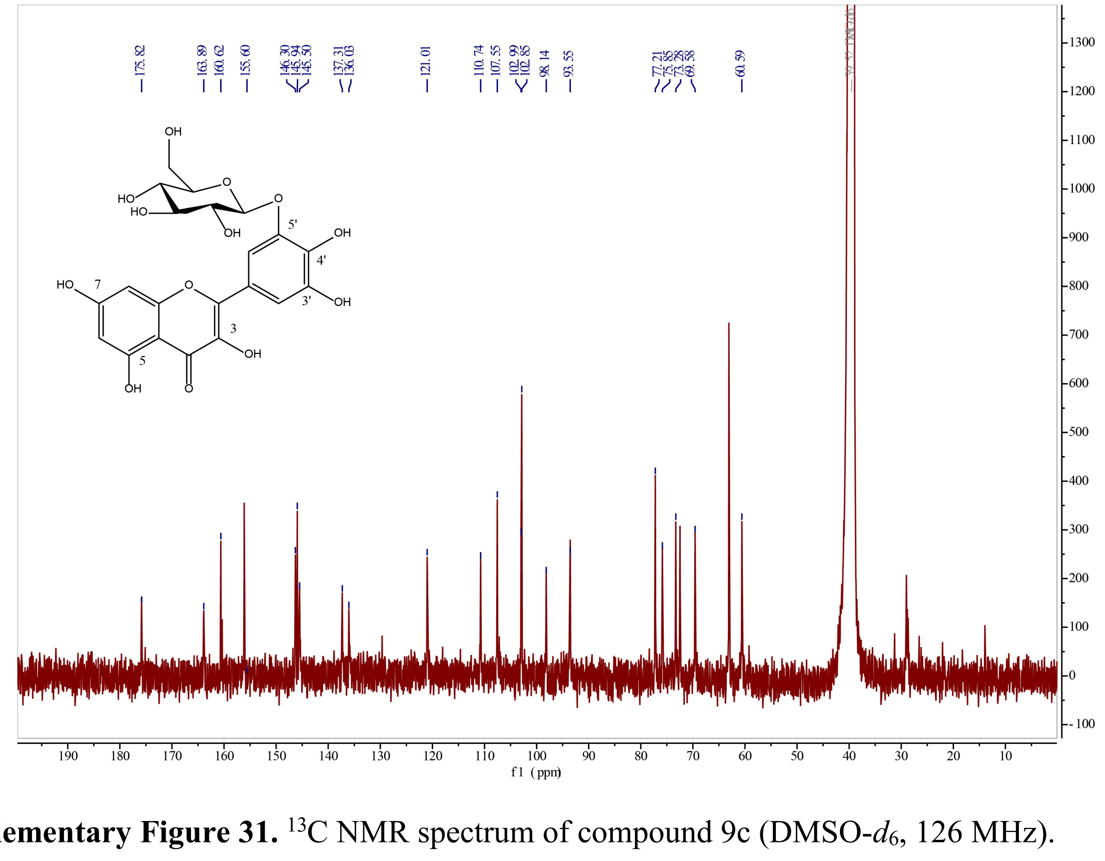

Supplementary Figure 31. ^{13}C NMR spectrum of compound 9c (DMSO-d_6, 126 MHz).

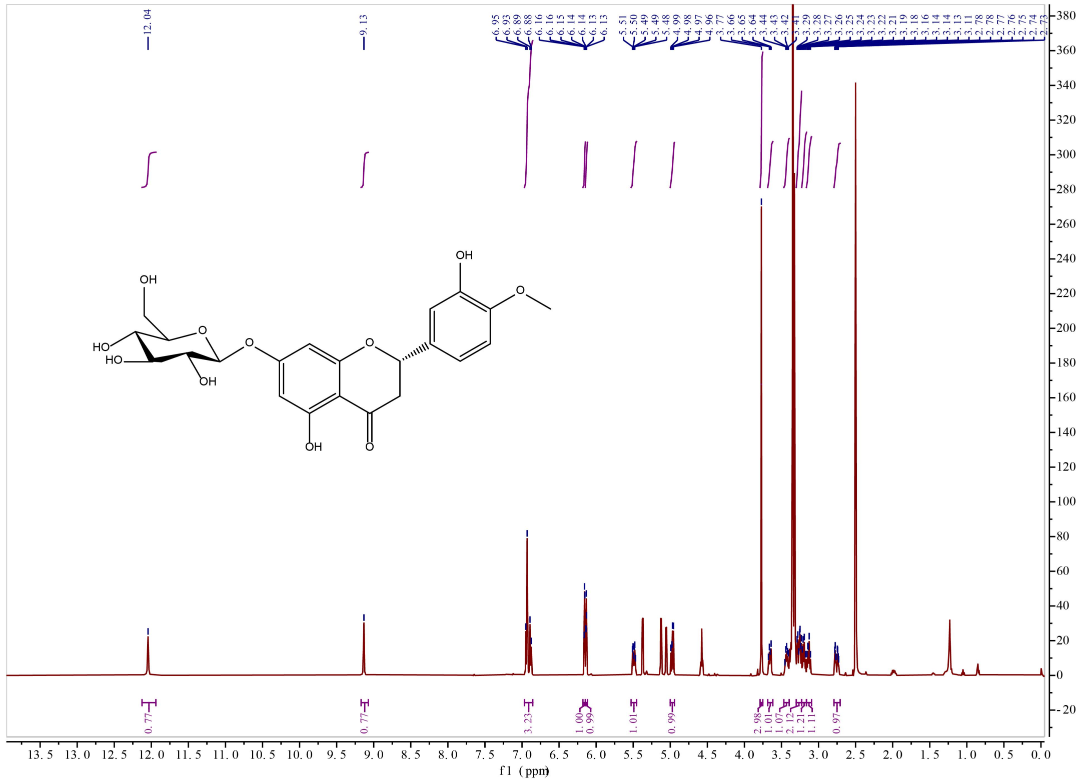

Supplementary Figure 32. ^{1}H NMR spectrum of compound 10a (DMSO-d_6, 500 MHz).

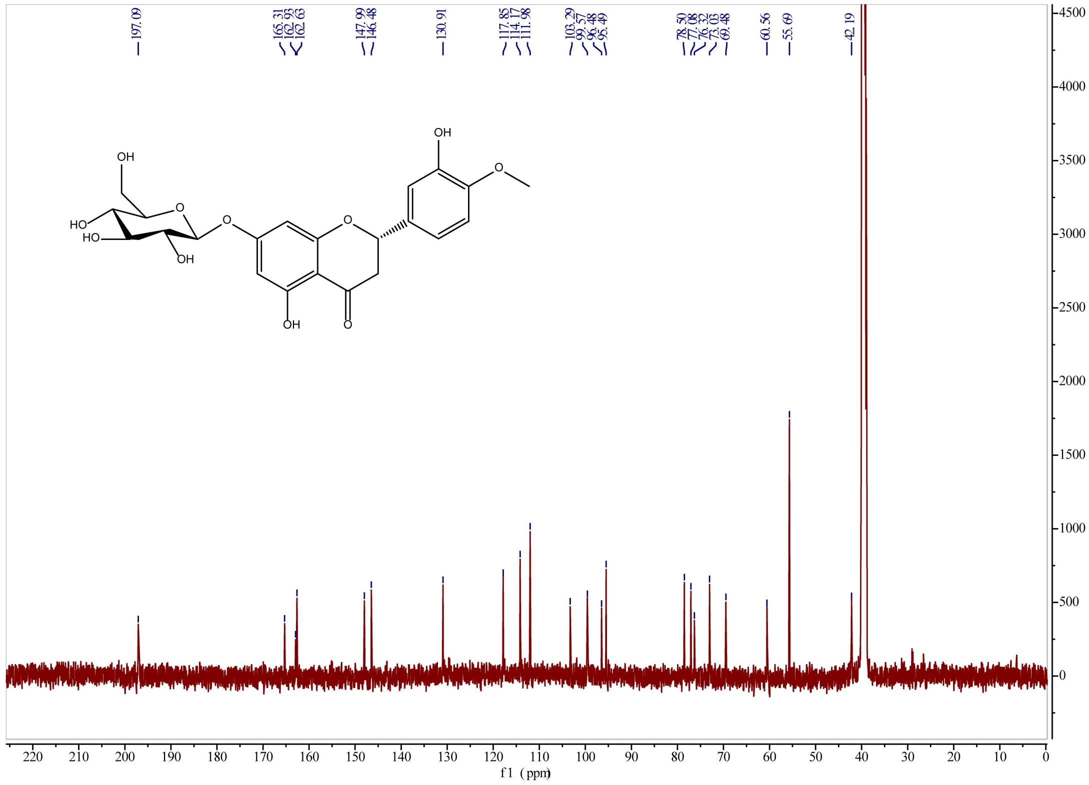

Supplementary Figure 33. ^{13}C NMR spectrum of compound 10a (DMSO-d_6, 126 MHz).

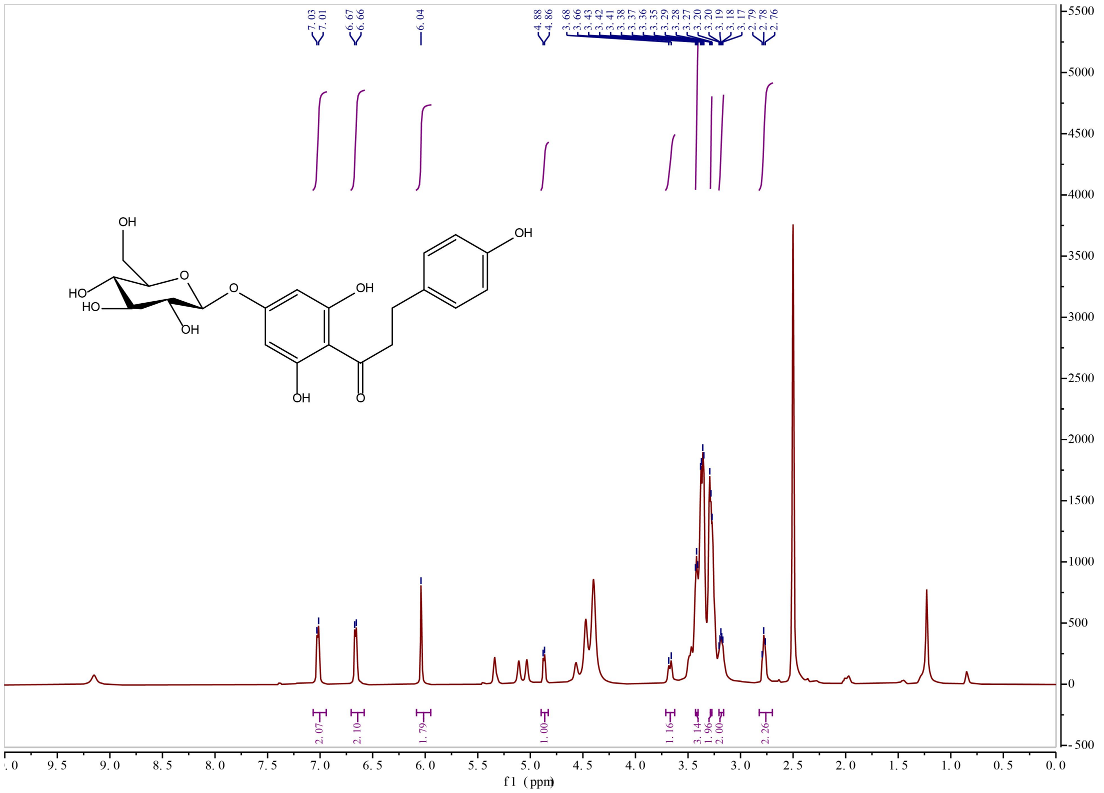

Supplementary Figure 34. ^{1}H NMR spectrum of compound 11a (DMSO-d_6, 500 MHz).

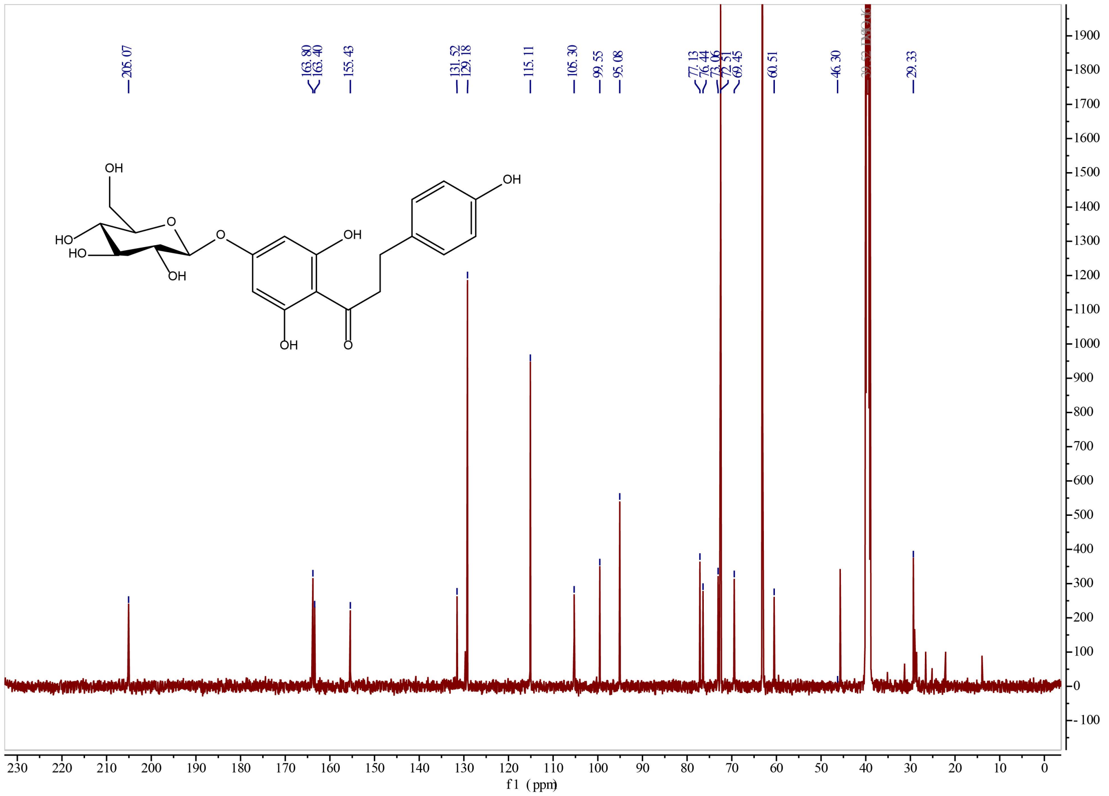

Supplementary Figure 35. ^{13}C NMR spectrum of compound 11a (DMSO-d_6, 126 MHz).

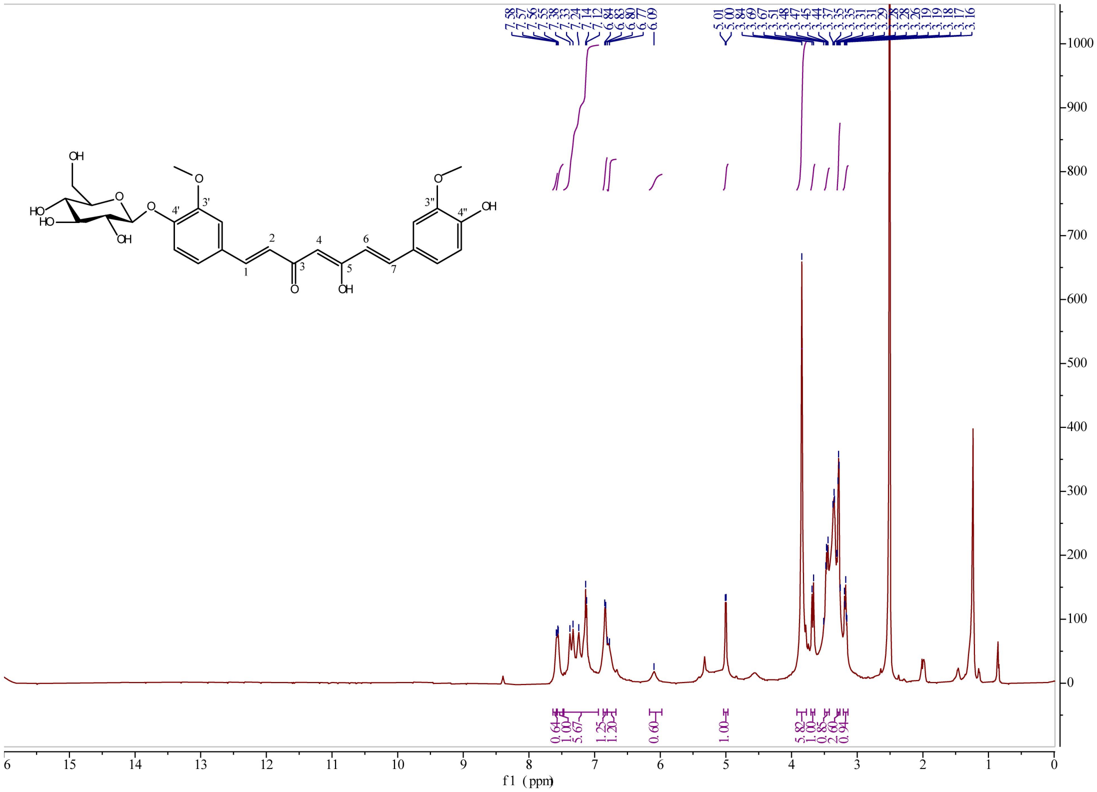

Supplementary Figure 36. ^{1}H NMR spectrum of compound 12b (DMSO-d_6, 500 MHz).

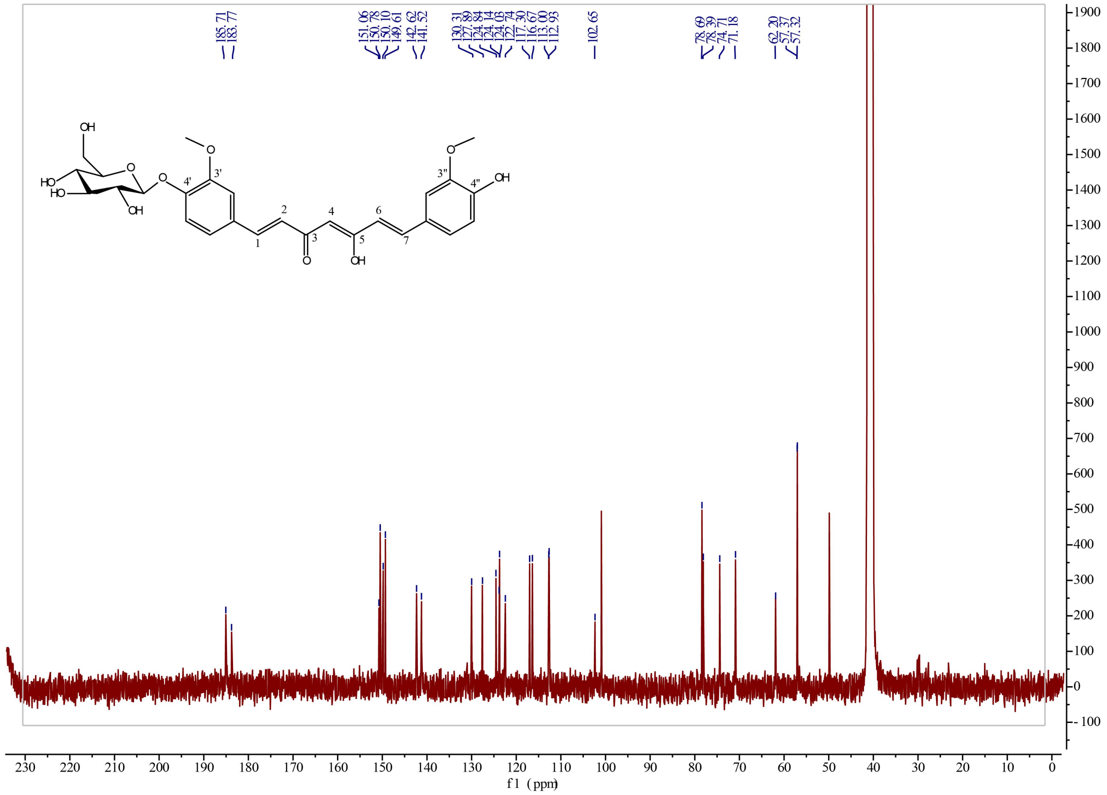

Supplementary Figure 37. ^{13}C NMR spectrum of compound 12b (DMSO-d_6, 126 MHz).
